# Peroxiredoxin 6 mediates protective function of astrocytes in Aβ proteostasis

**DOI:** 10.1186/s13024-020-00401-8

**Published:** 2020-09-09

**Authors:** Joanna E. Pankiewicz, Jenny R. Diaz, Mitchell Martá-Ariza, Anita M. Lizińczyk, Leor A. Franco, Martin J. Sadowski

**Affiliations:** 1 Department of Neurology, New York University Grossman School of Medicine, 550 First Avenue, Science Building, Room 10-07, New York, NY 10016 USA; 2 Department of Biochemistry and Molecular Pharmacology, New York University Grossman School of Medicine, New York, NY 10016 USA; 3 Department of Psychiatry, New York University Grossman School of Medicine, New York, NY 10016 USA

**Keywords:** Astrocytes, Alzheimer’s disease, β-Amyloid plaques, Microglia, Neurodegeneration, Peroxiredoxin 6, Plaque seeding, Proteostasis

## Abstract

**Background:**

Disruption of β-amyloid (Aβ) homeostasis is the initial culprit in Alzheimer’s disease (AD) pathogenesis. Astrocytes respond to emerging Aβ plaques by altering their phenotype and function, yet molecular mechanisms governing astrocytic response and their precise role in countering Aβ deposition remain ill-defined. Peroxiredoxin (PRDX) 6 is an enzymatic protein with independent glutathione peroxidase (Gpx) and phospholipase A2 (PLA_2_) activities involved in repair of oxidatively damaged cell membrane lipids and cellular signaling. In the CNS, PRDX6 is uniquely expressed by astrocytes and its exact function remains unexplored.

**Methods:**

*APPswe/PS1*_*dE9*_ AD transgenic mice were once crossed to mice overexpressing wild-type *Prdx6* allele or to *Prdx6* knock out mice. Aβ pathology and associated neuritic degeneration were assessed in mice aged 10 months. Laser scanning confocal microscopy was used to characterize Aβ plaque morphology and activation of plaque-associated astrocytes and microglia. Effect of *Prdx6* gene dose on plaque seeding was assessed in mice aged six months.

**Results:**

We show that hemizygous knock in of the overexpressing *Prdx6* transgene in *APP*_*swe*_*/PS1*_*dE9*_ AD transgenic mice promotes selective enticement of astrocytes to Aβ plaques and penetration of plaques by astrocytic processes along with increased number and phagocytic activation of periplaque microglia. This effects suppression of nascent plaque seeding and remodeling of mature plaques consequently curtailing brain Aβ load and Aβ-associated neuritic degeneration. Conversely, *Prdx6* haplodeficiency attenuates astro- and microglia activation around Aβ plaques promoting Aβ deposition and neuritic degeneration.

**Conclusions:**

We identify here PRDX6 as an important factor regulating response of astrocytes toward Aβ plaques. Demonstration that phagocytic activation of periplaque microglia vary directly with astrocytic PRDX6 expression level implies previously unappreciated astrocyte-guided microglia effect in Aβ proteostasis. Our showing that upregulation of PRDX6 attenuates Aβ pathology may be of therapeutic relevance for AD.

## Background

Alzheimer’s disease (AD) is a progressive neurodegenerative disease, where initially occurring β-amyloid (Aβ) deposition drives neurofibrillary pathology leading to synaptic and neuronal degeneration and gradual cognitive decline [1]. Containment of Aβ deposition or Aβ proteostasis is the initial defense mechanism preventing Aβ accumulation. Aβ plaques attract activated microglia, which are well recognized for their role in Aβ plaque transformation and containment. Microglia phagocytose diffuse Aβ peptide forming the plaque brim and compact Aβ within the plaque amyloid core [2] limiting extent of Aβ-associated neuritic degeneration [3]. AD-associated variants in Triggering Receptor Expressed in Myeloid cells 2 (TREM2) rendering microglia ineffective in Aβ plaque processing have been identified as second to the *APOE ε4* allele genetic risk factor for sporadic AD, highlighting the importance of periplaque glia function in Aβ proteostasis and in arresting downstream cascade of AD neurodegeneration [4]. Like microglia, activated astrocytes surround Aβ plaques and penetrate the plaques with their processes. However, in contrast to microglia the role of astrocytes in Aβ proteostasis and plaque formation remains ill-defined, mainly due to a paucity of known factors modulating astrocytic function in AD, and especially those, whose variable expression level would create a tractable experimental model. In this study, we decided to explore function of astrocytes in Aβ proteostasis and plaque formation through modulating expression level of an astrocytic native protein peroxiredoxin (PRDX) 6. PRDX6 is a dual function enzyme with independent glutathione peroxidase (Gpx) and phospholipase A2 (PLA_2_) activities, which is highly expressed by several cell lineages outside the CNS including alveolar epithelium, endothelium, and macrophages [5]. The PLA_2_ activity distinguishes PRDX6 from other peroxiredoxins and enables replacement of peroxidatively damaged cell membrane lipids, and cellular signaling [6, 7]. In the CNS, PRDX6 is expressed by astrocytes but no other type of glial cells [8, 9] and its exact function remains largely unexplored. In normal brain, expression of PRDX6 is dormant, while in AD it becomes selectively upregulated in astrocytes, which are associated with Aβ plaques and neurofibrillary tangles [9]. PRDX6 does not accumulate within the plaques, thus it is a reactive but not amyloid associated protein. To explore the function of PRDX6 in Aβ proteostasis we made *APP*_*swe*_*/PS1*_*dE9*_ transgenic (Tg) mice with hemizygous knock in of the overexpressing *Prdx6* transgene and those with *Prdx6* haplodeficiency. This experimental design showed that PRDX6 governs a protective function of astrocytes in countering Aβ deposition. We found an inverse dependence between *Prdx6* gene dose and Aβ plaque load and plaque-associated neuritic degeneration. *Prdx6* expression level also showed direct dependence with enticement of astrocytes and microglia to Aβ plaques, penetration of plaques by activated astrocytes, and phagocytic activation of periplaque microglia. Since PRDX6 is an astrocytic protein, the latter observation implies that astrocytes target microglia to Aβ plaques and circuitously regulate microglia dependent Aβ plaque processing.

## Methods

### Materials and reagents

Primers for polymerase chain reaction protocols were synthesized to order by Sigma-Aldrich (St. Louis, MO). Synthetic Aβ_1–40_ and Aβ_1–42_ peptides were produced by the ERI Amyloid Laboratory LLC (Oxford, CT) and handled as previously described [10, 11]. Antibody sources are individually listed below. All chemicals and reagents, were purchased from Sigma-Aldrich unless stated otherwise.

### Animals

**Fig. 1:**
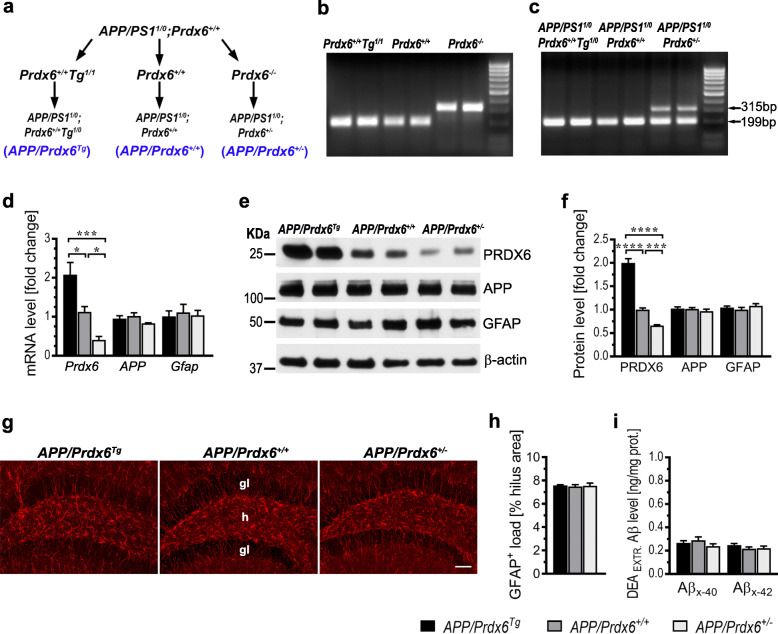
Development and characterization of *APP/Prdx6*^*Tg*^ and *APP/Prdx6*^*+/−*^ lines. **a** Breeding scheme: hemizygous *APP*_*SWE*_*/PS1*_*dE9*_ AD Tg mice (*APP/PS1*^*1/0*^*;Prdx6*^*+/+*^) were mated to mice, which on the background of endogenous *Prdx6* transgenically over-express wild type *Prdx6*^*129X1/SvJ*^ *allele* (*Prdx6*^*+/+*^*/Tg*^*1/1*^), wild type C57BL/6 J mice (*Prdx6*^*+/+*^) or *Prdx6* knock out mice (*Prdx6*^*−/−*^). Female F1 progeny of the following genotypes *APP/PS1*^*1/0*^*;Prdx6*^*+/+*^*Tg*^*1/0*^ (*APP/Prdx6*^*Tg*^), *APP/PS1*^*1/0*^*;Prdx6*^*+/+*^ (*APP/Prdx6*^*+/+*^), and *APP/PS1*^*1/0*^*;Prdx6*^*+/−*^ (*APP/Prdx6*^*+/−*^) were analyzed in this study. **b, c** Shown is *Prdx6* genotyping in parental and F1 generations, respectively. Wild type *Prdx6* PCR yields a single 199 bp band, whose variable intensity can side-to-side distinguish endogenous allele from transgenic overexpression, while 315 bp PCR product indicates transgenically disrupted *Prdx6* allele. Altered *Prdx6* expression does not affect mRNA and protein level for APP and GFAP as evidenced by qRT-PCR analysis in **d** and quantitative Western blotting in **e** and **f** in mice aged 4–6 weeks**,** respectively. Values in **d** and **e** express a fold change relative to *APP/Prdx6*^*+/+*^ line and represent mean (+SEM) from *n* = 6–8 mice per genotype in **d** and *n* = 5–7 mice per genotype in **f**. Altered *Prdx6* expression does not affect load of astrocytes before the onset of Aβ deposition. **g** Representative microphotographs of coronal cross-sections through the dorsal hippocampus from 3-month-old female mice of indicated genotypes immunolabeled against GFAP. **h** Quantification of GFAP^+^ astrocyte load in the dentate hilus. Values represent mean (+SEM) from n = 5 mice per genotype. **i** ELISA analysis of soluble (DEA-extractable) Aβ_x-40_ and Aβ_x-42_ levels in the brain cortex of mice aged 2 months. Values represent mean (+SEM) from *n* = 7–10 animals per genotype. *p =* 0.0008 for *Prdx6* mRNA in **d** and *p <* 0.0001 for PRDX6 protein in **f** (ANOVA); *p < 0.05, ***p < 0.001, and ****p < 0.0001 (Holm-Sidak’s post-hoc test). ANOVA for *APP* and *Gfap* mRNA in **d**, APP and GFAP protein in **f**, GFAP^+^ astrocyte load in **h**, and Aβ_x-40_ and Aβ_x-42_ levels in **i** is not significant. Abbreviations: gl - granular layer, h - hilus. Scale bar: 150 μm in **g**

All mouse care and experimental procedures were approved by Institutional Animal Care and Use Committees of the New York University Grossman School of Medicine. All transgenic mouse lines and C57BL/6 J wild type mice were obtained from The Jackson Laboratories (Bar Harbor, ME). *APP*_*swe*_*/PS1*_*dE9*_ AD model mice (B6.Cg-Tg(APPswe,PSEN1dE9)85Dbo/Mmjax; stock # 34832-JAX) express under the control of the mouse prion protein promoter the mouse amyloid precursor protein (APP) harboring the human Aβ sequence with the double Swedish familial AD mutation K594M/N595L and human presenilin 1 (PS1) with exon 9 deletion (dE9). In this model, the entire *APP*_*swe*_*/PS1*_*dE9*_ transgene is transmitted as a single Mendelian locus [12, 13]. *Prdx6* transgenic mice (C57BL/6 J-Tg(Prdx6)153Pgn/Pgn; stock #005902) overexpress the *Prdx6*^*129/SvJ-Tg*^ transgene containing the wild type *Prdx6* 129X1/SvJ allele, typical of the atherosclerosis-resistant 129/SvJ mouse strain, on the endogenous C57BL/6 J *Prdx6* expression background [14–16]. *Prdx6* knock out mice (B6.129-Prdx6tm1Pgn/Pgn; stock #005974) feature disruption of exons 1 and 2 of the *Prdx6* gene using a targeting vector containing the neomycin resistance and lacZ genes [17, 18]. All three transgenic mouse lines have been maintained on C57BL/6 J strain background. Hemizygous *APP*_*swe*_*/PS1*_*dE9*_ mice (*APP/PS1*^*1/0*^*;Prdx6*^*+/+*^*)* were mated with mice homozygous for *Prdx6*^*129/SvJ-Tg*^ transgene (*Prdx6*^*+/+*^*/Tg*^*1/1*^), wild type C57BL/6 J mice (*Prdx6*^*+/+*^), or with *Prdx6* knock out mice (*Prdx6*^*−/−*^). This cross-breeding strategy depicted in Fig. 1a, produced the following genotypes, which were analyzed in this study: *APP/PS1*^*1/0*^*;Prdx6*^*+/+*^*/Tg*^*1/0*^ (thereafter referred as *APP/Prdx6*^*Tg*^), *APP/PS1*^*1/0*^*;Prdx6*^*+/+*^ (thereafter referred as *APP/Prdx6*^*+/+*^), and *APP/PS1*^*1/0*^*;Prdx6*^*+/−*^ (thereafter referred as *APP/Prdx6*^*+/−*^). Littermates, which did not positively genotype for the *APP*_*swe*_*/PS1*_*dE9*_ transgene, were excluded from the study. Mice separated by sex were aged to 6 or 10 months in germ free vivarium with 12-h light/dark cycle and free access to food and water. Mouse health was assessed bi-weekly following standards of good husbandry practice [19]. Each examination started with observation of animals’ behavior in their home cages with attention paid to spontaneous activity, nest building, interaction with cage mates, and general appearance. This was followed by hands-on assessment of hydration status, postural reflexes, mobility, coat integrity, and search for subcutaneous anomalies including masses.

### Isolation and genotyping of genomic DNA

Genomic DNA was obtained by tail biopsy and genotyped for *APP*_*swe*_*/PS1*_*dE9*_ and *Prdx6*. Tail tissue was digested overnight at 55° C in the lysis buffer composed of 50 mM Tris-HCl (pH 8.0), 50 mM ethylenediaminetetraacetic acid (EDTA), 50 mM NaCl, 0.5% of N-Lauroyl sarcosine, 0.06% Tween 20, and 0.5 mg/mL of Proteinase K (Roche Life Science, Indianapolis, IN). Digestion product was centrifuged at 9000 x g for 10 min. DNA was precipitated from resulting supernatant with 5 volumes of 100% ethanol, trice washed with 70% ethanol, and dried up in a 55° C heat block. DNA pellets were resuspended in 80–120 μl of PCR grade dH_2_O and DNA concentration was photometrically determined using Nanodrop 2000 (Thermo Fisher Scientific, Waltham, MA). *APP*_*swe*_*/PS1*_*dE9*_ transgene was identified using real time polymerase chain reaction (RT-PCR) as previously described [10] on a CFX96™ Real-Time System (Bio-Rad Laboratories, Hercules, CA). *Prdx6* DNA was amplified using specific primers forward 1: 5′-CAGGATGGAGCCTCTATGCC-3′, forward 2: 5′-TGGCTTCTGAGACGGAAAGAA-3′, and reverse: 5′-CTTTGAACAGAACCAGGCAGG-3′ [18]. The starting amount of genomic DNA was always 500 ng. The PCR protocol was initiated by a 2-min denaturation at 94° C, followed by 40 amplification cycles consisting of 30 s denaturation at 92° C, 30 s annealing at 55° C, and 30 s extension at 70° C, and it was concluded by the final extension step at 72° C for 3 min. Amplified DNA was electrophoresis-separated on a 2% agarose gel for 60 min, visualized with 0.5 μg/ml ethidium bromide and imaged using Gel Doc XR+ (Bio-Rad Laboratories) (Fig. 1 b, c).

### Euthanasia and tissue handling

Animals were euthanized with an intraperitoneal injection of sodium pentobarbital (150 mg/kg) and transcardially perfused with 10 mM phosphate-buffered saline (PBS) (pH 7.4) with addition of heparin (1000 units/L). Immediately after perfusion brains were removed from the skulls and divided along the longitudinal fissure into left and right hemispheres. The left hemisphere was dissected under operating microscope on ice to isolate the brain cortex and the hippocampus, which were weighted, flash frozen in a dry ice/methanol cooling bath, and stored at -80° C until further analyses. The right brain hemisphere was fixed by immersion in 4% paraformaldehyde diluted in 100 mM phosphate buffer (PB) for 72 h at 4° C and stored in a mixture of 20% dimethyl sulfoxide and 20% glycerol in 0.01 PBS (pH 7.4) at 4° C until sectioning and immunohistochemistry.

### RNA isolation, reverse transcription, and quantitative real time PCR (qRT-PCR) analysis

RNA was extracted from the brain cortex of 4 to 6 weeks old mice immediately after their perfusion using RNeasy Mini Kit (Qiagen Sciences Inc., Germantown, MD). The RNA extract was treated with 2 U of DNAse I (Qiagen Sciences Inc.) and its purity was assessed by electrophoresing 1 μg of RNA on a 1.2% agarose gel for 60 min, staining with 0.5 μg/ml of ethidium bromide and imaging using Gel Doc XR+. Five-hundred nanograms of extracted RNA were reverse transcribed into cDNA using the iScript™ Advanced cDNA Synthesis Kit (Bio-Rad Laboratories). qRT-PCR was performed using SYBR® Green JumpStart™ Taq ReadyMix™ and the following primer sequences: 5′-TTG ATG ATA AGG GCA GGG AC-3′ forward and 5′-CTA CCA TCA CGC TCT CTC CC-3′ reverse for *Prdx6* [18], 5′-CGA ACC CTA CGA AGA AGC CAC-3′ forward and 5′-GCT TTC TGG AAA TGG GCA TGT TC-3′ reverse for *APP* [20], 5′-GCT TTC TGG AAA TGG GCA TGT TC-3′ forward and 5′-TCTGCCTCCAGCCTCAGGTT-3′ reverse for *Gfap* [21], and 5′-CCG GGG CTG GCA TTG CTC TC-3′ forward and 5′-TGT TGG GGG CCG AGT TGG GA-3′ reverse for murine glyceraldehyde-3-phosphate dehydrogenase (*Gapdh*) [20]. Amplification efficiency for each primer pair was verified from the regression slope between the cDNA dilution log values and the averaged cycle threshold (C_T_) value obtained for each cDNA dilution. Expression of target genes: *Prdx6*, *APP*, and *Gfap* was analyzed using 2^−ΔΔCt^ method [22]. Firstly, C_T_ values for each target gene were normalized to the C_T_ values for *Gapdh* as the reference gene for each animal individually. Resulting ΔC_T_ values were then normalized to averaged ΔC_T_ values for a given target gene in *APP/Prdx6*^*+/+*^ line. Target gene expression in *APP/Prdx6*^*Tg*^ and *APP/Prdx6*^*+/−*^ lines was calculated as a fold difference relative to these in *APP/Prdx6*^*+/+*^ line. qRT-PCR was performed on a CFX96™ Real-Time System.

### Western blot analysis

Samples of the brain cortex stored at -80° C were thawed, weighted, and homogenized (1:10 wt/vol at 4° C) in a buffer consisting of 20 mM Tris-HCL (pH 7.4), 250 mM sucrose, 1 mM egtazic acid, 1 mM EDTA and 10 μg/mL of a Complete Protease Inhibitor Cocktail (Roche Life Science) supplemented with 1 mM phenylmethylsulfonyl fluoride, and leupeptin, antipain, and pepstatin (5 μg/mL each). The three-step homogenization procedure included manual trituration in a pestle grinder, passing the tissue through a 28-gauge needle, and final sonication. Cellular debris were cleared by 3 min centrifugation at 10,000×g and 4° C and protein concentration in the resulting supernatant was measured by bicinchoninic acid (BCA) method using Pierce™ BCA Protein Assay Kit (Thermo Fisher Scientific), according to the manufacturer provided manual. Aliquots of brain homogenates containing 20 μg of the total protein for PRDX6 and APP and 5 μg for GFAP and β-actin were resolved on 10% SDS-PAGE and electroblotted onto nitrocellulose membranes, which were then blocked with 5% nonfat milk and incubated with the following primary antibodies anti-PRDX6 (rabbit polyclonal 1:10,000; Abcam Inc., Cambridge, MA), anti-GFAP (rabbit polyclonal 1:10,000; DAKO, Denmark), anti-APP (mouse monoclonal, clone C1/6.1 1:3000; BioLegend, San Diego, CA), and anti-β-actin (mouse monoclonal 1:10,000, Sigma-Aldrich). The antigen–antibody complexes were detected using horseradish peroxidase conjugated sheep anti-mouse or anti-rabbit secondary antibodies (1:30,000; GE Healthcare Bio-Sciences Corp. Pittsburgh, PA) and visualized using SuperSignal West Pico PLUS Chemiluminescent Substrates (Thermo Fisher Scientific). Resulting autoradiograph were digitized into a 600 dpi TIFF files and analyzed using NIH ImageJ v1.52a software (Bethesda, MD).

### Biochemical analyses of Aβ_x − 40_ and Aβ_x − 42_ levels

Samples of brain cortex homogenate were prepared analogously to those for the Western blot analysis and subjected to diethylamine (DEA) or formic acid (FA) extractions [23, 24], which release soluble or total Aβ peptides, respectively. Concentration of Aβ_x − 40_ and Aβ_x − 42_ in either fraction was determined on ELISA plates coated with HJ2 (anti-Aβ_35–40_) or HJ7.4 (anti-Aβ_37–42_) mouse monoclonal antibodies (0.5 μg/well; gift of Dr. DM Holtzman), respectively [20, 25]. Biotinylated 4G8 mouse monoclonal antibody (anti-Aβ_17–24_,1:5000; BioLegend) [26] was used as the detection antibody. Optic densities of ELISA readouts were converted to the actual concentrations of Aβ peptides based on the standard curves prepared from FA-treated synthetic Aβ_1–40_ and Aβ_1–42_ peptides. Final Aβ_x-40_ and Aβ_x-42_ concentrations in the brain tissue were reported in reference to the protein concentration considering all dilutions made during DEA and FA extractions [25].

### Histology and immunohistochemistry

The right brain hemisphere was serially sectioned into coronal 40-μm-thick sections using a freezing microtome (Leica Microsystems, Wetzlar, Germany). The sections were alternately collected into 10 series, then randomly selected for the following stainings: 1) Thioflavin-S (ThS), 2) anti-Aβ (mouse monoclonal HJ3.4 directed against the N-terminus of Aβ, 1:100; gift of Dr. DM Holtzman) [20, 25, 27]; 3) anti-GFAP (mouse monoclonal 1:2000; Sigma-Aldrich); 4) anti-Iba1 (rabbit polyclonal 1:1000; Wako Chemicals Inc., Richmond VA); 5) anti-CD68 (rabbit polyclonal 1:200, Abcam Inc., Cambridge, MA), and 6) Gallyas silver staining. For the anti-Aβ immunostaining the sections were pretreated with 44% FA for 10 min to enhance antigen availability [25] and the immunohistochemistry protocol was conducted using peroxidase M.O.M. and 3,3′-diaminobenzidine kits from Vector Laboratories (Burlingame, CA) as per manufacturer manual. For anti-GFAP immunostaining biotinylated horse-anti mouse IgG secondary antibody (1:500; Vector Laboratories) was used, while for anti-CD68 and anti-Iba1 immunostainings the secondary antibody was biotinylated goat anti-rabbit IgG (1:500; Vector Laboratories). The immunostaining protocol was concluded with streptavidin conjugated Cy3 fluorochrome (1:1000; Vector Laboratories) and Th-S counterstaining to visualize relationship between glial cells and fibrillar plaques as previously described [27]. Gallyas silver staining was carried out at 10° C and it was modified from its original protocol [26, 28] by extending the time of alkaline silver iodide enucleation to 40 min.

Specifically, for this project we developed several multi-fluorochrome labeling protocols to interrogate the relationship between Aβ plaques and plaque-associated glia cells. The fibrillar plaque core was labeled first with X-34 (10 μM in 40% ethanol [pH 10]) and then various glial antigens were immunostained with one of the following antibodies: anti-GFAP (rabbit polyclonal 1:2000; Dako/Agilent Technologies; Santa Clara, CA), anti-Iba1 (rabbit polyclonal 1:800; Wako Chemicals USA Inc.) or anti-CD68 (rat monoclonal 1:200; Abcam Inc.). Prior to applying the primary antibody, non-specific staining was blocked with a mixture of 10% normal goat serum, 1% bovine serum albumin (BSA), and 0.3% Triton X-100 in 10 mM PBS (pH 7.4) and in the case of anti-CD68 immunostaining additionally Avidin/Biotin Blocking Kit (Vector Laboratories) was used as per manufacturer manual. The secondary antibodies were Texas Red conjugated goat anti-rabbit IgG (1:500; Vector Laboratories) or goat biotinylated anti-rat IgG (1:500; Abcam Inc.) followed by Cy3 conjugated streptavidin (1:500). Subsequently, we immunolabelled the diffuse (non-fibrillar) Aβ within the plaque brim with HJ3.4 mouse monoclonal antibody (1:100; gift of Dr. D.M. Holtzman) followed by Dylight 488 conjugated horse anti-mouse IgG secondary antibody (1:500; Vector Laboratories). Cell nuclei were optionally stained with 1 μM of DRAQ5 (Thermo Fisher Scientific) as the fourth and the final step of the protocol.

For TREM2 visualization the sections were first pretreated with 10 mM sodium citrate and 0.5% Tween 20 (pH 6.0) for 20 min at 85° C to help the antigen retrieval [29]. Following labeling of the fibrillar plaque core with X-34, non-specific immunostaining was blocked with 5% normal donkey serum (Jackson ImmunoResearch, West Grove, PA) and 0.3% Triton X-100. The primary antibodies were anti-TREM2 (sheep polyclonal 1:250; R&D Systems, Minneapolis MN) and anti-Iba1 (rabbit polyclonal 1:1000; Wako Chemicals Inc.), while the secondary antibodies were donkey anti-sheep Alexa Fluor 594 conjugated (1:500; Thermo Scientific Scientific) and donkey anti-rabbit Alexa Fluor 488 conjugated (1:500; Jackson ImmunoResearch) both diluted in 4% normal donkey serum and 0.3% Triton, respectively. For analysis of cellular localization of PRDX6, the sodium citrate antigen retrieval protocol was used too. The primary antibodies were either anti-GFAP (mouse monoclonal 1:1,500; Sigma-Aldrich) or anti-Iba1 (mouse monoclonal 1:100; Sigma-Aldrich) and anti-PRDX6 (rabbit polyclonal 1:250; Abcam Inc.), while the secondary antibodies were goat anti-mouse Alexa Fluor 488 conjugated (1:500; Jackson ImmunoResearch) and goat anti-rabbit Alexa Fluor 594 conjugated (1:500; Jackson ImmunoResearch), respectively. For analysis of Aβ plaque-associated axonal dystrophy antigen retrieval with sodium citrate was used as well. The primary antibodies were anti-Aβ_1–16_ (rabbit polyclonal 1:500; BioLegend) and anti-Neurofilament (mouse monoclonal [clone SIM 312] 1:300; BioLegend), while the secondary antibodies were goat anti-rabbit Alexa Fluor 488 conjugated (1:500; Jackson ImmunoResearch) and goat anti-mouse Alexa Fluor 594 conjugated (1:500; Jackson ImmunoResearch), respectively.

### Quantitative analysis of Aβ plaque load and that of astrocytes and microglia

The load is defined as the percentage of a cross-sectional profile covered by positively-thresholded objects. It was determined using whole-section approach, where the entire cross-sectional profile of a given anatomical structure is digitally photographed and thresholded to prevents sampling bias [27]. We quantified the load of Th-S labeled fibrillar Aβ plaques and that of HJ3.4 immunostained Aβ plaques both in the brain cortex and in the hippocampus and loads of GFAP^+^ astrocytes and Iba1^+^ and CD68^+^ microglia in the brain cortex. Cortical load was analyzed on 3 coronal sections per brain taken at the approximated levels of the anterior commissure, the rostral portion of the hippocampus, and the mammillary bodies, while the hippocampal load was analyzed on 4–5 serial sections spaced 800 μm apart. Load values obtained from individual cortical and hippocampal profiles were averaged for each brain. Infrequent Th-S or anti-Aβ positive vascular profiles were edited out from digitized images. Sections immunolabelled for GFAP, Iba1 and CD68 also were counterstained with Th-S and the glia cell load was indexed to that of Th-S^+^ plaques on the same section. All load analyses were performed using NIH ImageJ v1.52. In addition, the load of GFAP^+^ cells was quantified in the dentate hilus in mice aged 2 months before the onset of Aβ deposition to assess for differences resulting from variable *Prdx6* expression. Numerical density of neuritic plaques was determined by taking their count on 3 cortical and 4–5 hippocampal profiles per brain on Gallyas stained sections and dividing obtained count by combined area of cross-sectional profiles. Similarly, we quantified the numerical density of nascent plaques in the brain cortex of mice aged 6 months.

### Laser scanning confocal microscopy (LSCM) and segmentation analysis of Aβ plaques

Images of multi-fluorochrome labeled Aβ plaques were taken under immersion oil, 63x, and 1.4 N.A. objective and 2x digital zoom using Zeiss LSM 880 microscope and ZEN Black 2.3 SP1 acquisition software v. 14.0.18.201 (Carl Zeiss AG; Oberkochen, Germany). Z stacks of 0.5-μm-thick serial tomograms with 25% overlap (increased to 50% for TREM2 imaging) were acquired through the entire thickness of Aβ plaques. For morphometric analysis 8 serial tomograms (or 12 for TREM2 imaging) dissecting through the center of the plaque fibrillar core were taken out from the original Z-stack and collapsed into a 2D image. Monochromatic images of individual plaque labels were then converted to 8-bit format, contrast enhanced, and analyzed using NIH ImageJ v1.52 (Fig. S1a-c). The following metrics of Aβ plaques were analyzed: 1) plaque area (the cross-sectional area of Aβ plaque implicit within the outline of HJ3.4 immunolabel [Fig. S1b]), 2) plaque/core ratio (ratio between the plaque area and the area of its X-34^+^ fibrillar core), 3) GFAP plaque index (GFAP^+^ % plaque area) (Fig. S1c), 4) density of plaque-associated microglia (number of Iba1^+^ microglia cells implicit within the Aβ plaque outline divided by the outline area), 5) Iba1 plaque index (Iba1^+^ % plaque area), 6) microglia barrier robustness index (Iba1/X-34% co-localization), 7) CD68 phagosome plaque index (CD68^+^ % plaque area), 8) TREM2 plaque area (TREM2^+^ % area within the 40 μm radius from the plaque center), 9) TREM2 to Iba1 ratio in plaque-associated microglia, 10) number of NF-positive swellings per plaque, and 11) NF plaque index (NF^+^ % plaque area).

### Statistical analysis

Normal distribution of data was confirmed first using the Kolmogorov-Smirnov and Shapiro-Wilk tests. Differences across multiple data sets were analyzed using one-way analysis of variance (ANOVA) followed by Holm-Sidak’s multiple comparison test to ascertain significance between individual data set pairs. Statistical analysis was performed using GraphPad Prism v 7.03 (GraphPad Software Inc. San Diego, CA). All data presented in the manuscript are given as the mean and the standard error of the mean (SEM). Magnitude of change across genotypes was always expressed as the percentage value for *APP/Prdx6*^*+/+*^ mice, or as a fold-change when direct comparison between *APP/Prdx6*^*Tg*^ and *APP/Prdx6*^*+/−*^ genotypes was made.

## Results

### Overexpression of *Prdx6* reduces Aβ load while *Prdx6* haplodeficiency increases it

To differentially express *Prdx6* we crossed *APP*_*SWE*_*/PS1*_*dE9*_ (*APP/Prdx6*^*+/+*^) AD Tg model mice [12, 13] with mice transgenically over-expressing *Prdx6*^*129X1/SvJ*^ *allele* on the wild-type *Prdx6* background (*Prdx6*^*+/+*^*/Tg*^*1/1*^) [15] or with *Prdx6* knock out (*Prdx6*^*−/−*^) mice [18]. Resulting lines were termed *APP/Prdx6*^*Tg*^ and *APP/Prdx6*^*+/−*^, respectively (Fig. 1a-c); and exhibited no phenotypical or gross behavioral defects or precocious morbidity compared to parental *APP/Prdx6*^*+/+*^ mice. Levels of *Prdx6* mRNA and PRDX6 protein in the brain cortex of mice aged 4–6 weeks show 2:1:0.6 ratio across *APP/Prdx6*^*Tg*^, *APP/Prdx6*^*+/+*^, and *APP/Prdx6*^*+/−*^ genotypes, while the expression of APP and GFAP remain unchanged both by mRNA and protein analysis (Fig. 1d-f). Likewise, we found no differences in the morphology of GFAP^+^ astrocytes and their load in the dentate hilus, which we analyzed in mice aged 3 months (Fig. 1g, h). Soluble Aβ_x-40_ and Aβ_x-42_ peptides were extracted using diethyalamine [DEA] from the brain cortex of mice aged 2 months and quantified using C-terminus specific ELISAs and their levels showed no significant differences across the genotypes (Fig. 1i). These data evidence that alteration of *Prdx6* gene expression does not affect resting state of astrocytes or brain Aβ levels prior to the onset of plaque formation.

**Fig. 2:**
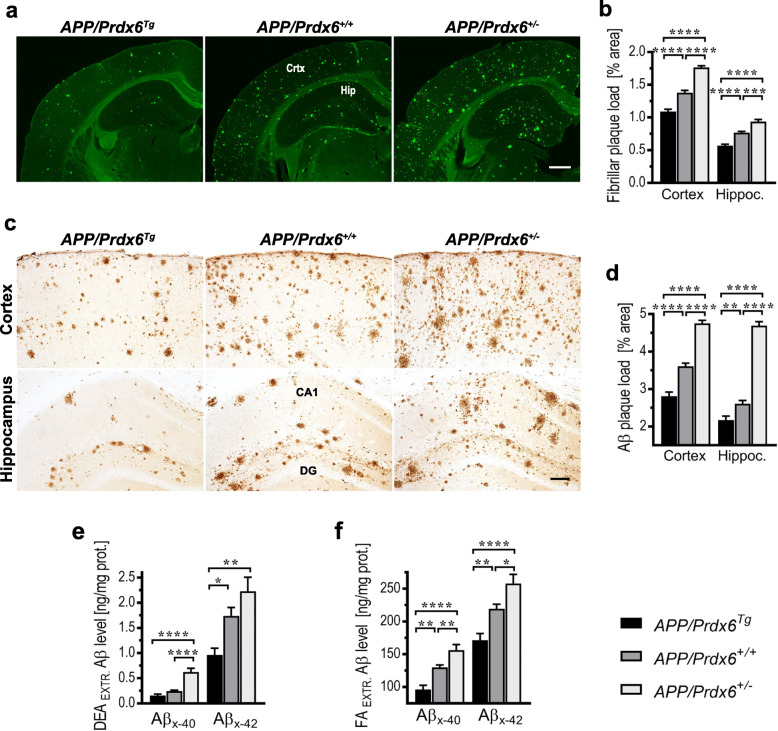
Aβ load in APP_SWE_/PS1_dE9_ mice varies inversely with *Prdx6* gene expression level. **a** and **c** Representative microphotographs of coronal cross-sections through the somatosensory cortex and the dorsal hippocampus from 10-month-old female mice of indicated genotypes, which were stained for fibrillar Aβ plaques with Thioflavin-S or immunolabeled with HJ3.4 clone directed against the N-terminus of Aβ peptide, respectively. Quantitative analysis of fibrillar **b** and immunopositive **d** Aβ plaque load in the brain cortex and in the hippocampus evidencing suppression of Aβ deposition in *Prdx6* overexpressing mice and increased Aβ deposition in *Prdx6* haplodeficient mice. Values represent mean (+SEM) from *n* = 9–12 female mice per genotype. **e** and **f** ELISA analysis of soluble (DEA-extractable) and total (FA-extractable) Aβ_x-40_ and Aβ_x-42_ levels in the brain cortex of female 10-month-old mice, respectively. Values represent mean (+SEM) from n = 7–13 animals per genotype. *p <* 0.0001 in **b**, **d**, **e**, and **f** (ANOVA); *p < 0.05, **p < 0.01, ***p < 0.001, and ****p < 0.0001 (Holm-Sidak’s post-hoc test). Abbreviations: CA1 – cornu Ammonis sector 1, Crtx – cortex, DG – dentate gyrus, Hip – hippocampus. Scale bars: 750 μm in **a** and 50 μm in **c**

The effect of *Prdx6* gene dose on Aβ deposition was detailed in female mice aged 10 months. *APP/Prdx6*^*Tg*^ line shows 21% reduction in the fibrillar (Th-S^+^) Aβ plaque load in the brain cortex (*p <* 0.0001) and 26% reduction in the hippocampus (*p <* 0.0001) compared to *APP/Prdx6*^*+/+*^ controls, while conversely *APP/Prdx6*^*+/−*^ mice demonstrate 28% (*p <* 0.0001) and 22% (*p <* 0.001) increase in respective structures (Fig. 2a, b). The load of immunolabeled Aβ plaques shows 22% reduction in the brain cortex (*p <* 0.0001) and 17% reduction in the hippocampus (*p <* 0.01) in *APP/Prdx6*^*Tg*^ mice compared to *APP/Prdx6*^*+/+*^ controls, while in *APP/Prdx6*^*+/−*^ mice it is increased by 32% (*p <* 0.0001) and 80% (*p <* 0.0001) (Fig. 2c, d), respectively. In direct comparison with *APP/Prdx6*^*Tg*^ line, APP*/Prdx6*^*+/−*^ mice show 1.6-fold higher load of fibrillar Aβ plaques both in the brain cortex and in the hippocampus (*p <* 0.0001), while the load of immunolabeled Aβ plaque is 1.7-fold higher in the cortex (*p <* 0.0001) and 2.2-fold higher in the hippocampus (*p <* 0.0001).

Levels of soluble and total deposited Aβ_x-40_ and Aβ_x-42_ were quantified in DEA and FA brain cortex extracts, respectively. In *APP/Prdx6*^*Tg*^ mice soluble Aβ_x-40_ and Aβ_x-42_ levels are reduced by 35% (*p =* 0.18) and 45% (*p <* 0.05) compared to *APP/Prdx6*^*+/+*^ controls, respectively; while in *APP/Prdx6*^*+/−*^ mice they are increased by 156% (*p <* 0.0001) and 28% (*p =* 0.09), respectively (Fig. 2e). Total Aβ_x-40_ and Aβ_x-42_ levels are reduced by 26% (*p <* 0.01) and 22% (*p <* 0.01) in *APP/Prdx6*^*Tg*^ mice compared to *APP/Prdx6*^*+/+*^ controls*,* respectively; while in *APP/Prdx6*^*+/−*^ mice they are increased by 20% (*p <* 0.01) and 18% (*p <* 0.05), respectively (Fig. 2f). In direct comparison with *APP/Prdx6*^*Tg*^ line, APP*/Prdx6*^*+/−*^ mice show 3-fold (*p <* 0.0001) and 1.3-fold (*p <* 0.01) higher levels of soluble Aβ_x-40_ and Aβ_x-42_, respectively; and 1.6-fold (*p <* 0.0001) and 1.5-fold (*p <* 0.01) higher levels of total Aβ_x-40_ and Aβ_x-42_, respectively. Of note, *Prdx6* haplodeficiency effects an increase in Aβ accumulation in AD Tg mice comparable to that caused by *Trem2* haplodeficiency or *Trem2 R47H* mutant, conferring loss of TREM2 function [29].

To examine whether the effect of *Prdx6* gene dose on Aβ deposition is gender-specific, we compared Aβ plaque load in female and male littermates at the age of 10 months (Fig. S1 a-d). Significant reduction in Aβ plaque load in *APP/Prdx6*^*Tg*^ line and its increase in *APP/Prdx6*^*+/−*^ line compared to *APP/Prdx6*^*+/+*^ controls is evident in both sexes. When matched for *Prdx6* genotype, female mice show significantly higher load of plaques than male mice, irrespective of plaque type and brain structure analyzed. This latter observation is consistent with reports indicating gender-based disparity in Aβ plaque load in some of AD Tg mouse models, with propensity toward greater Aβ accumulation in females [30, 31]. All further analyses presented in this study were performed on female mice to assure gender consistence.

### *Prdx6* gene dose does not influence Aβ-associated astrocytic load but has a direct relationship with that of microglia

**Fig. 3:**
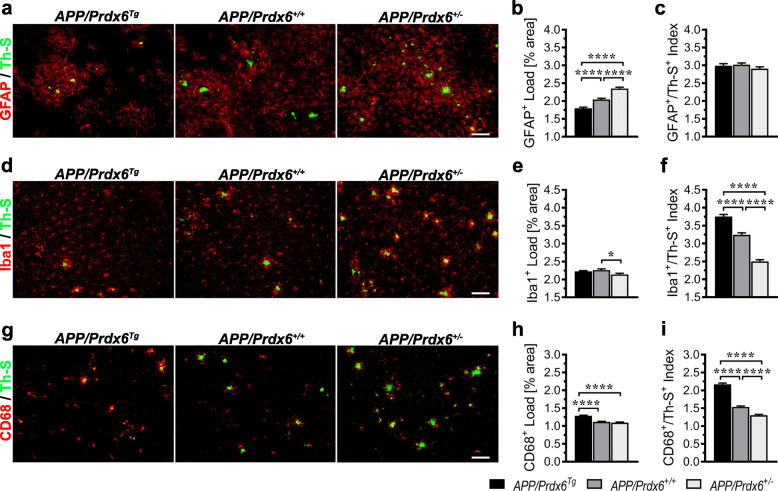
*Prdx6* gene dose does not affect global response of astrocytes to Aβ deposition but shows a direct relationship with that of microglia. **a**, **d**, **g** Representative microphotographs of anti-GFAP, −Iba1, or -CD68 immunolabeled coronal sections through the somatosensory cortex from 10-month-old female mice of indicated genotypes, respectively. Counterstaining with Thioflavin-S (Th-S) reveals fibrillar plaques to which the load of glial cells was indexed. **b**, **e**, and **h** Quantitative analysis of GFAP^+^, Iba1^+^, and CD68^+^ cell load, in the brain cortex, respectively. **c**, **f**, and **i** Show loads of GFAP^+^, Iba1^+^, and CD68^+^ cells indexed to these of Th-S^+^ fibrillar plaques, respectively. While Th-S^+^-indexed GFAP^+^ cell load demonstrates no statistically significant differences across the genotypes, those for Iba^+^ and CD68^+^ cells have significant direct relationship with *Prdx6* gene dosage. All values represent mean (+SEM) from *n* = 12 mice per genotype. *p <* 0.0001 in **b**, **f**, **h**, and **i**, *p =* 0.27 in **c** and *p =* 0.0142 in **e** (ANOVA); *p < 0.05, and ****p < 0.0001 (Holm-Sidak’s post-hoc test). Scale bar: 50 μm in **a**, **d**, and **g**

To gain insight into the mechanism(s) underlying opposing relationship between *Prdx6* gene dose and Aβ deposition and inform design of subsequent experiments we quantified loads of GFAP^+^ astrocytes and Iba1^+^ and CD68^+^ microglia in the cortex of female mice aged 10 months and indexed them to the load of fibrillar (Th-S^+^) Aβ plaques. The unindexed GFAP^+^ load in *APP/Prdx6*^*Tg*^ mice shows 12% (*p <* 0.0001) reduction, while in *APP/Prdx6*^*+/−*^ mice 15% increase (*p <* 0.0001) compared to *APP/Prdx6*^*+/+*^ controls (Fig. 3a, b). The differences across genotypes equalize and become statistically insignificant when GFAP^+^ load is indexed to that of Th-S^+^ plaques (Fig. 3c). Unindexed loads of Iba1^+^ and CD68^+^ microglia vary only modestly across the genotypes: with 5% reduction (*p <* 0.05) in the Iba1^+^ load in *APP/Prdx6*^*+/−*^ mice vs. *APP/Prdx6*^*+/+*^ controls (Fig. 3d, e), and 16% increase (*p <* 0.0001) in the CD68^+^ load in *APP/Prdx6*^*Tg*^ mice vs. *APP/Prdx6*^*+/+*^ controls (Fig. 3g, h). However, when either Iba1^+^ or CD68^+^ load becomes indexed to that of Th-S^+^ plaques, a strong gradient of microglia activation across the three genotypes emerges: *APP/Prdx6*^*Tg*^ > *APP/Prdx6*^*+/+*^ *> APP/Prdx6*^*+/−*^ (Fig. 3f, i). The Th-S^+^ indexed Iba1^+^ load is increased by 16% (*p <* 0.0001) in *APP/Prdx6*^*Tg*^ mice, while in *APP/Prdx6*^*+/−*^ mice it is reduced by 23% (*p <* 0.0001) compared to *APP/Prdx6*^*+/+*^ controls. Analogously, the Th-S^+^ indexed CD68^+^ load is increased by 41% (*p <* 0.0001) in *APP/Prdx6*^*Tg*^ mice and reduced by 15% (*p <* 0.0001) in *APP/Prdx6*^*+/−*^ mice compared to *APP/Prdx6*^*+/+*^ controls. In direct comparison with *APP/Prdx6*^*Tg*^ line, APP*/Prdx6*^*+/−*^ mice show 1.5-fold (*p <* 0.0001) and 1.7-fold (*p <* 0.0001) reduction in the loads of Iba1^+^ and CD68^+^ microglia cells, when these are indexed to that of Th-S^+^ plaques. These findings collectively indicate that neither *Prdx6* overexpression effects hyperactive astrogliosis nor *Prdx6* haplodeficiency impairs ability of astrocytes to mount response to Aβ deposition since the GFAP^+^ load remains proportional to that of fibrillar amyloid deposits across the three genotypes. Unexpectedly, we find that activation of microglia varies directly with the *Prdx6* gene dose and this includes CD68 antigen expression, which reflects phagocytic microglia activity.

### *Prdx6* overexpression and haplodeficiency produce opposite effects on Aβ plaque modeling by plaque-associated astrocytes and microglia

**Fig. 4:**
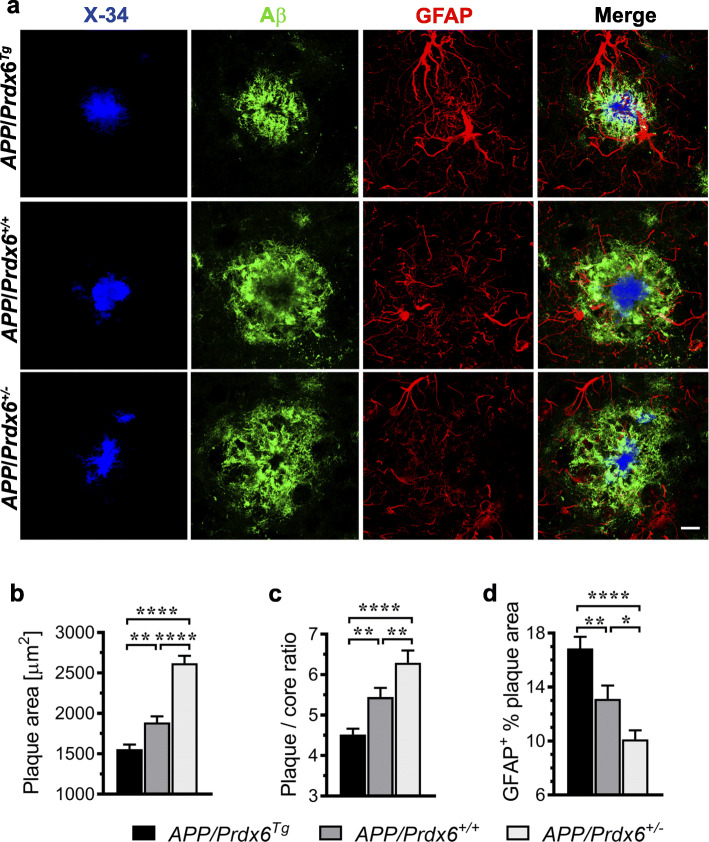
*Prdx6* expression alters plaque morphology and their penetrance by astrocytes. **a** Representative LSCM images of mature plaques triple labeled with X-34 (fibrillar core), anti-Aβ (HJ3.4 clone), and anti-GFAP antibodies from female mice of indicated genotypes demonstrating inverse relationship between *Prdx6* gene dose and size of Aβ plaques and degree of plaque compactness expressed as a ratio between cross-sectional areas of Aβ plaque label and X-34 labeled fibrillar core. *Prdx6* overexpression also is associated with increased penetrance of Aβ plaques by astrocytes, while in *Prdx6* haplodeficient mice the number and caliber of plaque penetrating GFAP positive processes are diminished. Shown are quantitative analyses of: average Aβ plaque cross-sectional area **b**, plaque/core ratio **c**, and GFAP plaque index (GFAP^+^ % plaque area) **d**. Values in **b** and **c** represent mean (+SEM) from *n* = 70–90 randomly selected plaques per genotype, while those in **d** from *n* = 30 randomly selected plaques per genotype. *p <* 0.0001 in **b**, **c**, and **d** (ANOVA); *p < 0.05, **p < 0.01, and ****p < 0.0001 (Holm-Sidak’s post-hoc test). Scale bar: 10 μm in **a**

We used multi-antigen labeling and laser scanning confocal microscopy (LSCM) to analyze effects of *Prdx6* gene dose on the morphology of Aβ plaques and behavior of plaque-associated glial cells (Fig. S2 a-c). In *APP/Prdx6*^*Tg*^ mice, the average plaque area (i.e. area implicit within the outline of anti-Aβ label) is reduced by 18% (*p <* 0.01) compared to *APP/Prdx6*^*+/+*^ controls, while the ratio of plaque area to the area of its X-34^+^ fibrillar core (plaque/core ratio) is reduced by 17% (*p <* 0.01) evidencing increased plaque compactness (Fig. 4a-c). Opposite effects are seen in APP*/Prdx6*^*+/−*^ mice, where the average plaque area and the plaque/core ratio are increased by 39% (*p <* 0.0001) and 16% (*p <* 0.01) compared to *APP/Prdx6*^*+/+*^ controls, respectively. In direct comparison with *APP/Prdx6*^*Tg*^ line, in APP*/Prdx6*^*+/−*^ mice an average Aβ plaque area is 1.7-fold bigger (*p <* 0.0001) and has 1.4-fold higher (*p <* 0.0001) plaque/core ratio. These changes in Aβ plaque morphology are closely associated with differential behavior of plaque-associated astrocytes and microglia discernible across the genotypes. In *APP/Prdx6*^*Tg*^ mice, there is increased infiltration of Aβ plaques by GFAP^+^ astrocytic processes, reflected by 28% increase in the GFAP plaque index (GFAP^+^ % plaque area) vs. *APP/Prdx6*^*+/+*^ controls (Fig. 4a, d). Conversely, in *APP/Prdx6*^*+/−*^ mice Aβ plaques are characterized by rarefaction of astrocytic processes and show 23% reduction (*p <* 0.05) in the GFAP plaque index vs. *APP/Prdx6*^*+/+*^ controls. In direct comparison with *APP/Prdx6*^*Tg*^ line, the GFAP plaque index in APP*/Prdx6*^*+/−*^ mice is reduced by 1.7-fold (*p <* 0.001). These observations evidence that *Prdx6* gene dose alters the way astrocytes engage Aβ plaques, rather than produces global effect on the astrocytic activation in response to Aβ deposition. In addition, we analyzed plaque content of apolipoprotein (apo) E, which in the CNS is produced by astrocytes and co-deposited with Aβ in the plaques [32]. In *APP/Prdx6*^*Tg*^ mice, there is 41% increase (*p <* 0.0001) in the apoE plaque index (ApoE^+^ % plaque area) compared to APP*/Prdx6*^*+/+*^ controls, while in *APP/Prdx6*^*+/−*^ mice that index is reduced by 26% (*p <* 0.0001) (Fig. S3 a, b). In direct comparison with APP*/Prdx6*^*+/−*^ line, *APP/Prdx6*^*Tg*^ mice show 1.9-fold higher (*p <* 0.0001) apoE plaque index reflecting increased engagement of Aβ plaques by astrocytes. We also assessed expression of complement component 3 (C3) by astrocytes engaging Aβ plaques by measuring the C3^+^ / GFAP^+^ plaque index (Fig. S4 a, b). This was done because elevated C3 expression is considered a marker of the neurotoxic astrocytic phenotype also known as A1 [33–35]. In *APP/Prdx6*^*Tg*^ mice the C3^+^ / GFAP^+^ index is reduced by 26.7% compared to *APP/Prdx6*^*+/+*^ controls (*p* < 0.0001), while in *APP/Prdx6*^*+/−*^ mice it is increased by 31.8% (*p* < 0.0001). In direct comparison with the *APP/Prdx6*^*Tg*^ line, plaque-associated astrocytes in APP*/Prdx6*^*+/−*^ mice show 1.8-fold higher C3 expression (*p <* 0.0001).

**Fig. 5:**
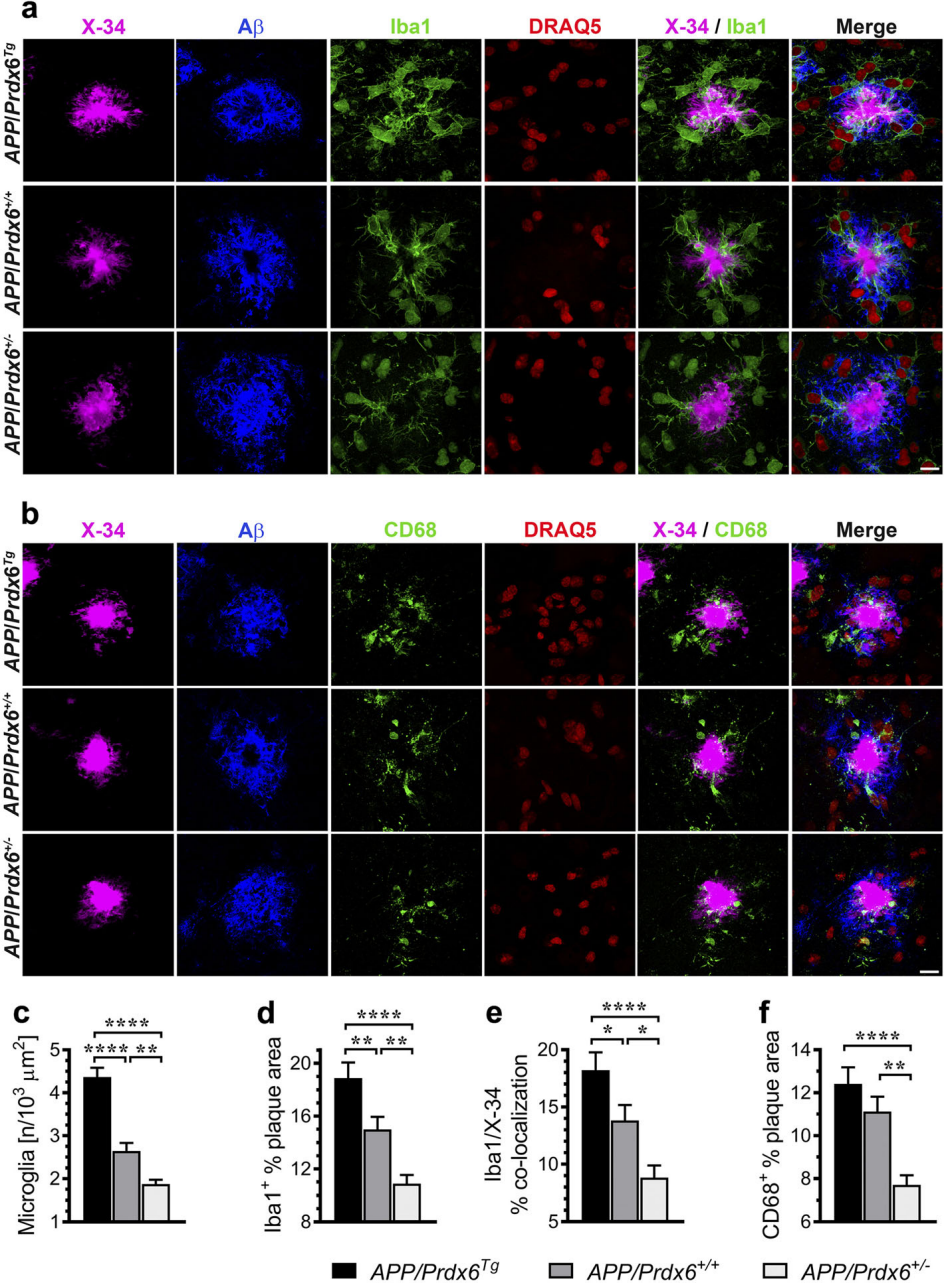
Phagocytic activation of plaque-associated microglia is enhanced in mice overexpressing *Prdx6* and attenuated in *Prdx6* haplodeficient mice. Representative LSCM images of mature plaques co-labeled with X-34 (fibrillar core), anti-Aβ (HJ3.4 clone), anti-Iba1, and DRAQ5 (nuclear stain) in **a**, and with X-34, anti-Aβ, anti-CD68, and DRAQ5 in **b** evidencing direct relationship between *Prdx6* gene dose and the number of periplaque microglia cells and expression of Iba1 and CD68 microglial activation markers. Shown are morphometric analyses of plaque-associated microglia number **c**, Iba1 plaque index (Iba1^+^ % plaque area) **d**, microglia barrier robustness (Iba1/X-34% co-localization) **e**, and CD68 phagosome plaque index (CD68^+^ % plaque area) **f**. Values in **c** through **f** represent mean (+SEM) from *n* = 26–35 randomly selected plaques per staining and per genotype. *p <* 0.0001 in **c** through **f** (ANOVA); *p < 0.05, **p < 0.01, and ****p < 0.0001 (Holm-Sidak’s post-hoc test). Scale bar: 10 μm in **a** and **b**

**Fig. 6:**
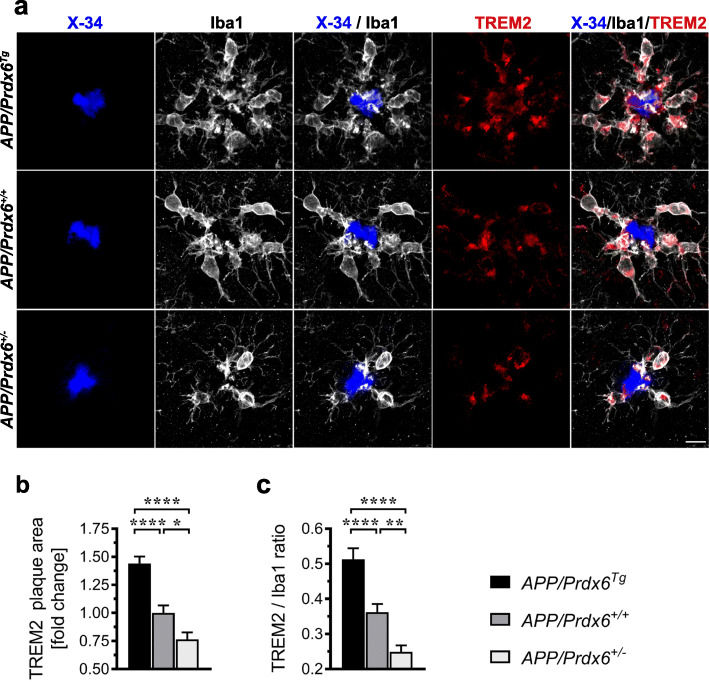
Expression of TREM2 in plaque-associated microglia shows direct relationship with *Prdx6* gene dose. **a** Representative LSCM images of mature plaques co-labeled with X-34 (fibrillar core), anti-Iba1, and anti-TREM2 antibodies from female animals of indicated genotypes evidence increased periplaque expression of TREM2 in *APP/Prdx6*^*Tg*^ line and conversely reduced expression in *APP/Prdx6*^*+/−*^ line compared to *APP/Prdx6*^*+/+*^ controls. Quantification of TREM2 immunoreactivity within surrounding of the X-34^+^ fibrillar core (TREM2 plaque area) **b** and TREM2 to Iba1 ratio in plaque-associated microglia **c**. Values represent mean (+SEM) from *n* = 45 randomly selected plaques per genotype. *p <* 0.0001 in **b** and **c** (ANOVA); *p < 0.05, **p < 0.01, and ****p < 0.0001 (Holm-Sidak’s post-hoc test). Scale bar: 10 μm in **a**

Along with the differences in the way how astrocytes engage Aβ plaques we also noticed significant differences in the behavior of plaque-associated microglial cells. In *APP/Prdx6*^*Tg*^ mice, there is 65% increase (*p <* 0.0001) in the number of plaque-associated Iba1^+^ microglia cells (Fig. 5a, c), 26% increase (*p <* 0.01) in the plaque Iba1 index (Iba1^+^ % plaque area) (Fig. 5d), 32% increase (*p <* 0.05) in the microglia barrier robustness index (Iba1/X-34% co-localization) (Fig. 5e), and 12% increase (*p =* 0.16) in the CD68 phagosome plaque index (CD68^+^ % plaque area) (Fig. 5b. f) compared to *APP/Prdx6*^*+/+*^ controls. *APP/Prdx6*^*+/−*^ mice display opposite changes in microglia activation metrics. The average number of plaque-associated Iba1^+^ microglia cells shows 29% reduction (*p <* 0.01), plaque Iba1 index 27% reduction (*p <* 0.01), microglia barrier robustness index 36% reduction (*p <* 0.05), and CD68 phagosome index 31% reduction (*p <* 0.01) vs. *APP/Prdx6*^*+/+*^ controls. In direct comparison with *APP/Prdx6*^*Tg*^ line, APP*/Prdx6*^*+/−*^ mice show 2.3-fold lesser number of periplaque Iba1^+^ cells (*p <* 0.0001), 1.7-fold lower plaque Iba1 index (*p <* 0.0001), 2.1-fold lower microglia barrier robustness index (*p <* 0.0001), and 1.6-fold lower CD68 phagosome index (*p <* 0.0001). There also is a striking effect of *Prdx6* gene dose on TREM2 expression by periplaque microglia. *APP/Prdx6*^*Tg*^ mice feature 44% increase (*p <* 0.0001) in the TREM2^+^ label of the plaque (TREM2 plaque area) (Fig. 6a, b) and 42% increase (*p <* 0.0001) in the TREM2/Iba1 ratio (Fig. 6c) reflecting upregulation of TREM2 expression on the individual microglial cell level compared to *APP/Prdx6*^*+/+*^ controls. In contrast, *APP/Prdx6*^*+/−*^ mice show down regulation of TREM2 expression, evidenced by 23% reduction (*p <* 0.05) in TREM2 plaque area and 31% reduction (*p <* 0.001) in TREM2/Iba1 ratio compared to *APP/Prdx6*^*+/+*^ controls. In direct comparison with *APP/Prdx6*^*Tg*^ line these metrics are reduced by 1.9-fold (*p <* 0.0001) and 2.1-fold (*p <* 0.0001) in APP*/Prdx6*^*+/−*^ mice, respectively. Thus, *Prdx6* gene dose shows direct relationship with phagocytic activation of periplaque microglia and inverse relationship with the size and compactness of Aβ plaques, what is consistent with function of periplaque microglia recognized for phagocytosing diffuse Aβ in the plaque brim and forming the plaque core. *Prdx6* overexpression has a protective effect by enhancing Aβ proteostasis, while *Prdx6* haplodeficiency to the contrary confers functional impairment of periplaque microglia in countering Aβ deposits akin to that rendered by TREM2 haplodeficiency or R47H *Trem2* mutant [3, 29, 36].

### PRDX6 protein is upregulated in plaque-associated astrocytes, but it is undetectable in microglia

**Fig. 7:**
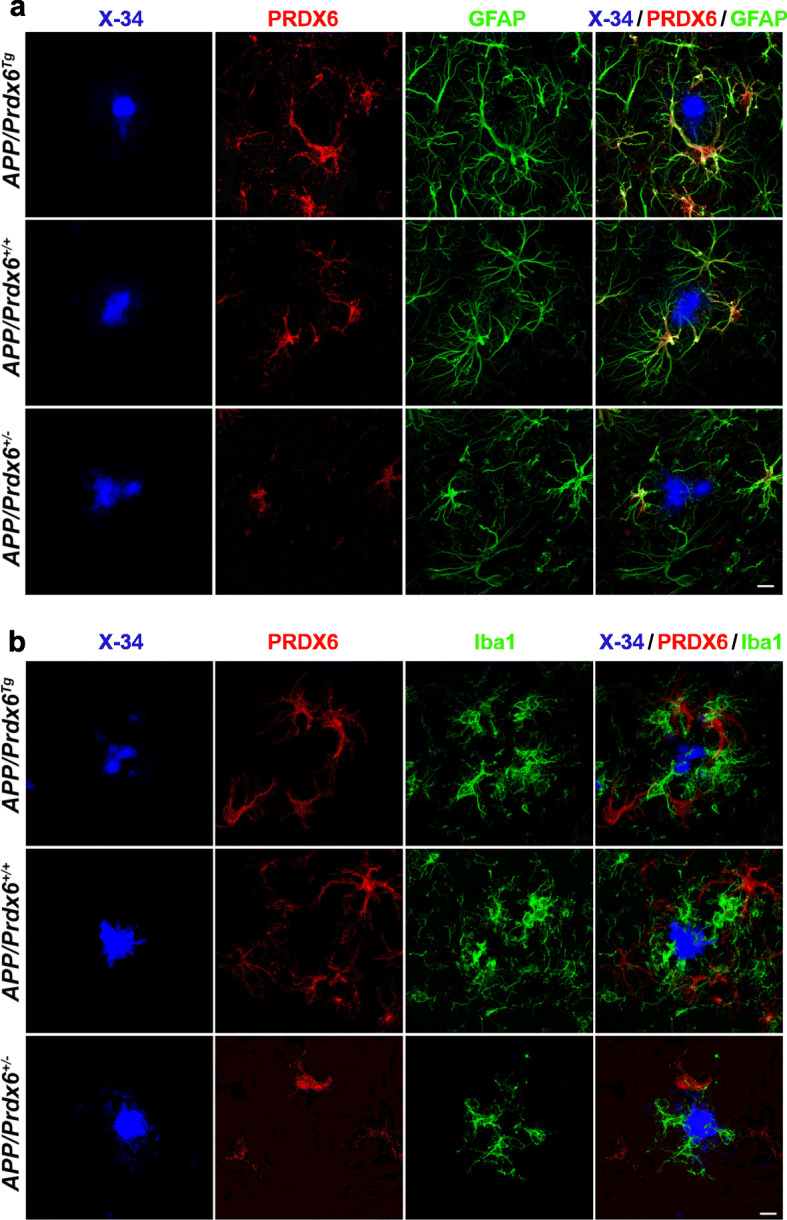
PRDX6 protein is exclusively expressed by astrocytes and absent in plaque-associated microglia. Representative LSCM images of mature plaques co-labeled with X-34 (fibrillar core), anti-PRDX6, and anti-GFAP antibodies in **a**, and with X-34, and anti-PRDX6, and anti-Iba1 antibodies in **b** evidencing colocalization of PRDX6 immunostaining with that of GFAP but not with Iba1. Co-labeling with anti-PRDX6, and anti-Iba1 antibodies in **b** reveals in fact two distinct cell populations localized further apart and closer to the fibrillar core, respectively. There is apparent *APP/Prdx6*^*Tg*^ > *APP/Prdx6*^*+/+*^ > *APP/Prdx6*^*+/−*^ gradient of PRDX6 immunostaining intensity across the genotypes. PRDX6 immunostaining does not co-localize with the X-34 labeled fibrillar plaque core. Scale bar: 10 μm in **a** and **b**

We used multi-antigen labeling and LSCM imaging to interrogate cellular localization of PRDX6 protein in plaque-associated glial cells. PRDX6 strictly co-localizes to GFAP^+^ astrocytes and its expression follows *APP/Prdx6*^*Tg*^ > *APP/Prdx6*^*+/+*^ > *APP/Prdx6*^*+/−*^ gradient across the genotypes (Fig. 7a). Plaque-associated astrocytes feature marked upregulation of PRDX6 expression in comparison to astrocytes in Aβ plaque free, 3-month-old mice (Fig. S5a, b). Redistribution of PRDX6 immunoreactivity from the soma to astrocytic processes is a striking feature of periplaque astrocytic activation. This is pronounced in *APP/Prdx6*^*Tg*^ mice, noticeable in *APP/Prdx6*^*+/+*^ mice, but absent in *APP/Prdx6*^*+/−*^ animals (Fig. S5b). Activated astrocytes in *APP/Prdx6*^*Tg*^ mice also appear to be more hypertrophied compared to those in *APP/Prdx6*^*+/−*^ mice, especially in respect to the processes penetrating plaques (Fig. 4a, Fig. S4a, Fig. S5b). Apart from astrocytes grouped around Aβ plaques, activated, PRDX6^+^ astrocytes also can be found alongside blood vessels in 10-month-old mice (Fig. S5c). Similarly, expression of PRDX6 by activated, perivascular astrocytes has been described in AD autopsy material, including vessels which do not exhibit overt cerebral amyloid angiopathy [9]. Akin to plaque-associated astrocytes, perivascular astrocytes also show differences in PRDX6 expression level across the genotypes, which are commensurate with *Prdx6* gene dose.

There is no evidence of PRDX6 expression by plaque-associated microglia. Anti-PRDX6 and anti-Iba1 immunostainings show no co-localization in any of studied genotypes. In fact, the X-34/PRDX6/Iba1 triple labeling reveals two strikingly different, non-overlapping populations of plaque-associated cells: Iba1^+^ activated microglia and PRDX6^+^ cells, which judging by their “spider-like morphology” and localization farther apart from the X-34^+^ fibrillar core, are likely plaque-associated astrocytes (Fig. 7b). In a supplementary experiment, we also examined *Prdx6* transcript in affinity-purified microglia cultures where we find its level to be merely 1% of that in cultured astrocytes, and not increasing following lipopolysaccharide stimulation (Fig. S6). Thus, confirmatory to previous work of us and others [8, 9] we endorse that PRDX6 protein is expressed by astrocytes but not by microglia and its level becomes upregulated during astrocytic activation in *Prdx6* gene dose-dependent manner. Our demonstration that manipulation of the expression level of an astrocytic inherent protein produces coextensive effects on periplaque microglia activation implies that astrocytes circuitously guide microglia dependent Aβ plaque processing.

### Seeding of nascent Aβ plaque is inhibited by *Prdx6* overexpression while promoted by haplodeficiency

**Fig. 8:**
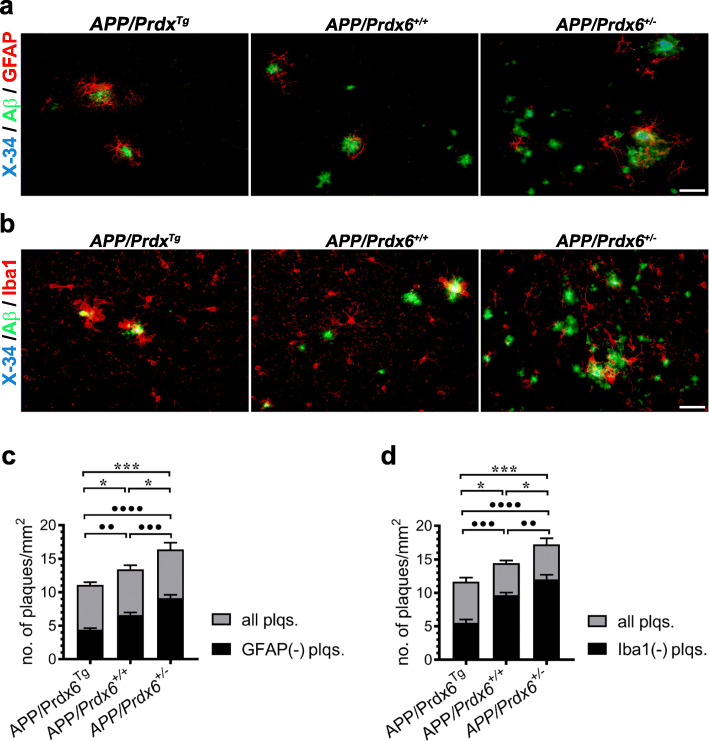
*Prdx6* expression modulates plaque seeding. Shown are representative images of nascent plaques in female mice aged 6 months of indicated genotypes, which were co-labeled with X-34 (fibrillar core), and anti-Aβ (HJ3.4 clone) and anti-GFAP antibodies in **a** and with X-34, anti-Aβ (HJ3.4 clone), and anti-Iba1 antibodies in **b** evidencing an inverse relationship between *Prdx6* expression level and the numerical density of nascent plaques and the numerical density of nascent plaques devoid of GFAP^+^ and Iba1^+^ cells. Quantitative analysis of nascent plaque numerical density in the brain cortex performed on X-34/Aβ/GFAP co-labeled sections in **c**, and on X-34/Aβ/Iba1 co-labeled sections in **d.** In this analysis nascent plaques were arbitrary defined as having no X-34^+^ core or having X-34^+^ core < 40 μm^2^. Black bars represent number of plaques devoid of GFAP^+^ or Iba1^+^ cells /mm^2^ and are superimposed on grey bars representing the total number of plaques /mm^2^ for a given genotype. Values in **c** and **d** represent mean (+SEM) from *n* = 6 female mice per genotype. *p <* 0.0001 in **c** and **d** (ANOVA); ^●●^*p <* 0.01, ^●●●^*p <* 0.001, and ^●●●●^*p <* 0.0001 denote significance for the density of GFAP and Iba1 devoid plaques across genotypes; *p < 0.05, and ***p < 0.001, denote significance for the density of all plaques (Holm-Sidak’s post-hoc test). Scale bar: 20 μm in **a** and **b**

Early stages of Aβ deposition remain only partially understood. Increasing concentration of soluble Aβ peptides is believed to drive Aβ self-assembly giving rise to precipitation of the earliest, immunodetectable deposits, which attract glial cells. To explore how variable *Prdx6* expression impacts Aβ plaque seeding we quantified numerical density of nascent plaques and their subsets devoid of GFAP^+^ or Iba1^+^ cells on X-34/Aβ/GFAP or X-34/Aβ/Iba1 triple-labeled sections, respectively. We define nascent plaques as circumscribed, immunopositive Aβ deposits having no X-34^+^ core or having X-34^+^ core with cross-sectional surface ≤40μm^2^ (Fig. 8a, b). This analysis was done in mice aged 6 months, representing an early stage of Aβ deposition in the *APP*_*swe*_*/PS1*_*dE9*_ Tg model [13]. In *APP/Prdx6*^*Tg*^ line the numerical density of all nascent plaques in the brain cortex shows 17% reduction (*p <* 0.05), while in APP*/Prdx6*^*+/−*^ mice 22% increase (*p <* 0.05) compared to *APP/Prdx6*^*+/+*^ controls (Fig. 8a-d). In comparison with *APP/Prdx6*^*Tg*^ line, 6-month-old APP*/Prdx6*^*+/−*^ mice have 1.5-fold (*p <* 0.001) higher density of nascent plaques. Numerical densities of plaques devoid of GFAP^+^ or Iba1^+^ cells also show significant differences across the genotypes. In *APP/Prdx6*^*Tg*^ mice numbers of astrocyte and microglia-free plaques are lesser by 33% (*p <* 0.01) and 43% (*p <* 0.001) compared to *APP/Prdx6*^*+/+*^ controls, respectively; while in APP*/Prdx6*^*+/−*^ mice they are increased by 38% (*p <* 0.001) and 24% (*p <* 0.01), respectively. In direct comparison between *APP/Prdx6*^*Tg*^ and APP*/Prdx6*^*+/−*^ lines, the latter shows 2.1-fold (*p <* 0.0001) and 2.2-fold (*p <* 0.0001) more of astrocyte and microglia-free plaques, respectively. We also detected significant differences in the phagocytic activity of microglia associated with nascent plaques across the mouse lines. This was determined by quantification of the CD68^+^ / X-34^+^ plaque index in a subset of nascent plaques, which already developed the amyloid core (Fig. S7a, b). *APP/Prdx6*^*Tg*^ mice exhibit 39.9% increase in the CD68^+^ / X-34^+^ plaque index compared to *APP/Prdx6*^*+/+*^ controls (*p* < 0.0001), while in *APP/Prdx6*^*+/−*^ mice the CD68^+^ / X-34^+^ index is reduced by 20.1% (*p* < 0.01). In direct comparison with *APP/Prdx6*^*Tg*^ line, APP*/Prdx6*^*+/−*^ mice show 1.8-fold lower CD68^+^ / X-34^+^ index (*p <* 0.0001).

In a complementary experiment, we quantified the load and numerical density of all Th-S^+^ plaques (nascent + mature) in the brain cortex and the hippocampus in mice aged 6 months and this analysis revealed *APP/Prdx6*^*Tg*^ < *APP/Prdx6*^*+/+*^ < *APP/Prdx6*^*+/−*^ rank order concerning both metrics with statistically significant differences across the genotypes for either anatomical structure (Fig. S8a-c). Taken together these findings indicate that activity level of astro- and microglia cells effectively impacts early stages of Aβ deposition and that this process is regulated by PRDX6.

### Plaque-associated axonal dystrophy varies inversely with *Prdx6* gene dose

**Fig. 9:**
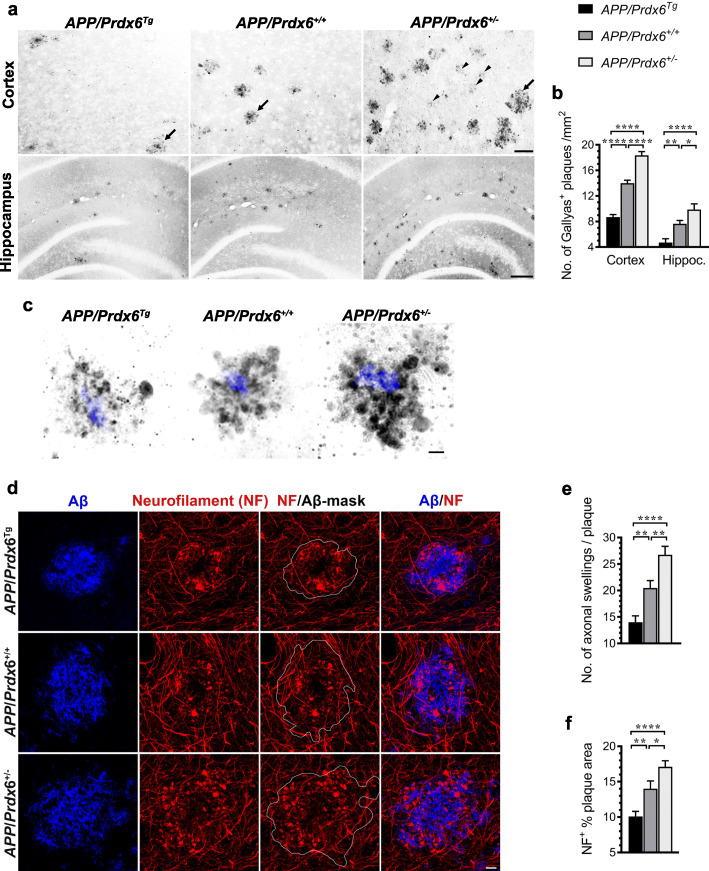
Neuritic degeneration is attenuated in *APP*_*SWE*_*/PS1*_*dE9*_ mice overexpressing *Prdx6* and exacerbated in *Prdx6* haplodeficient mice. **a** Shown are representative microphotographs of coronal cross-sections through the somatosensory cortex (upper panel) and the dorsal hippocampus (lower panel) from 10-month-old female mice of indicated genotypes, which were stained with Gallyas silver stain. Both the numerical density of neuritic plaques and the number and size of spheroid bodies forming the plaques increase in the rank order of *APP/Prd6*^*Tg*^ < *APP/Prd6*^*+/+*^ < *APP/Prd6*^*+/−*^. Arrows indicate mature plaques while arrowheads indicate early stage of neuritic degeneration associated with emerging plaques, which are abundant in the *APP/Prd6*^*−/−*^ mice. **b** Quantitative analysis of numerical density of Gallyas^+^ neuritic plaques in the brain cortex and in the hippocampus depicted as mean value (+SEM) from *n* = 6 female mice per genotype. **c** Shown are high magnification microphotographs of neuritic plaques produced by superimposing bright field picture of silver impregnated spheroids on fluorescent image of X-34 labeled fibrillar plaque core highlighting the differences in plaque composition across the genotypes, which include both the number and the distance spheroid spread away from the fibrillar core. **d** Representative LCMS images of plaques co-labeled with antibodies against the N-terminus of Aβ peptide (Aβ_1–16_) and neurofilaments (NF) from female mice of indicated genotypes demonstrating distorted trajectories of axons passing in the vicinity of the plaques and presence of axonal swellings (spheroids) in the area occupied by anti-Aβ immunolabeling (white encirclement indicated by Aβ plaque mask). Quantification of the number of NF^+^ axonal swellings per Aβ plaque in **e** and NF^+^ plaque index (NF^+^ % plaque area) in **f**. Values represent mean (+SEM) from *n* = 40 randomly selected plaques per genotype. *p <* 0.001 for the hippocampus in **b**, and *p <* 0.0001 for the brain cortex in **b**, **e**, and **f** (ANOVA); *p < 0.05, **p < 0.01, and ****p < 0.0001 (Holm-Sidak’s post-hoc test). Scale bars: 50 μm – the cortex and 200 μm the hippocampus in **a**, and 10 μm in **c** and **d**

Numerical density of neuritic plaques was quantified in female mice aged 10 months on section impregnated with Gallyas silver stain [25, 26, 28]. In *APP/Prdx6*^*Tg*^ mice, neuritic plaque density in the brain cortex and the hippocampus is reduced by 38% (*p <* 0.0001) and 39% (*p <* 0.01) compared to *APP/Prdx6*^*+/+*^ controls, respectively; while in APP*/Prdx6*^*+/−*^ mice conversely it shows 31% (*p <* 0.0001) and 29% (*p <* 0.05) increase, respectively (Fig. 9a, b). In comparison with *APP/Prdx6*^*Tg*^ line, the numerical density of neuritic plaques in APP*/Prdx6*^*+/−*^ mice is 2.1-fold higher in either the brain cortex or the hippocampus (*p <* 0.0001). There also are noticeable differences in the morphology of neuritic plaques across the genotypes. These concern the number of argentophilic spheroids and the distance they spread out from the plaque center following the *APP/Prdx6*^*Tg*^ < *APP/Prdx6*^*+/+*^ < *APP/Prdx6*^*+/−*^ rank order (Fig. 9a, c). Argentophilic spheroids represent focal swelling of axons, which degenerate passing through diffuse Aβ amassed around the plaque fibrillar core. They also are known to accumulate endosomal vesicles and neurofilaments [37], thus, we used double anti-Aβ/anti-neurofilament (NF) immunolabeling protocol to further characterize the impact of *Prdx6* gene dose on axonal dystrophy (Fig. 9d). *APP/Prdx6*^*Tg*^ mice show reduction in the average number of NF-positive swelling per plaque by 32% (*p <* 0.01) and in the NF plaque index (NF^+^ % plaque area) by 28% (*p <* 0.01) compared to *APP/Prdx6*^*+/+*^ controls, while in *APP/Prdx6*^*+/−*^ mice there is 31% (*p <* 0.01) and 28% (*p <* 0.01) increase in these metrics, respectively (Fig. 9e, f). In direct comparison with *APP/Prdx6*^*Tg*^ line, APP*/Prdx6*^*+/−*^ mice show 1.9-fold more of NF-positive swellings per plaque (*p <* 0.0001) and 1.7-fold higher NF plaque index (*p <* 0.0001).

## Discussion

**Fig. 10:**
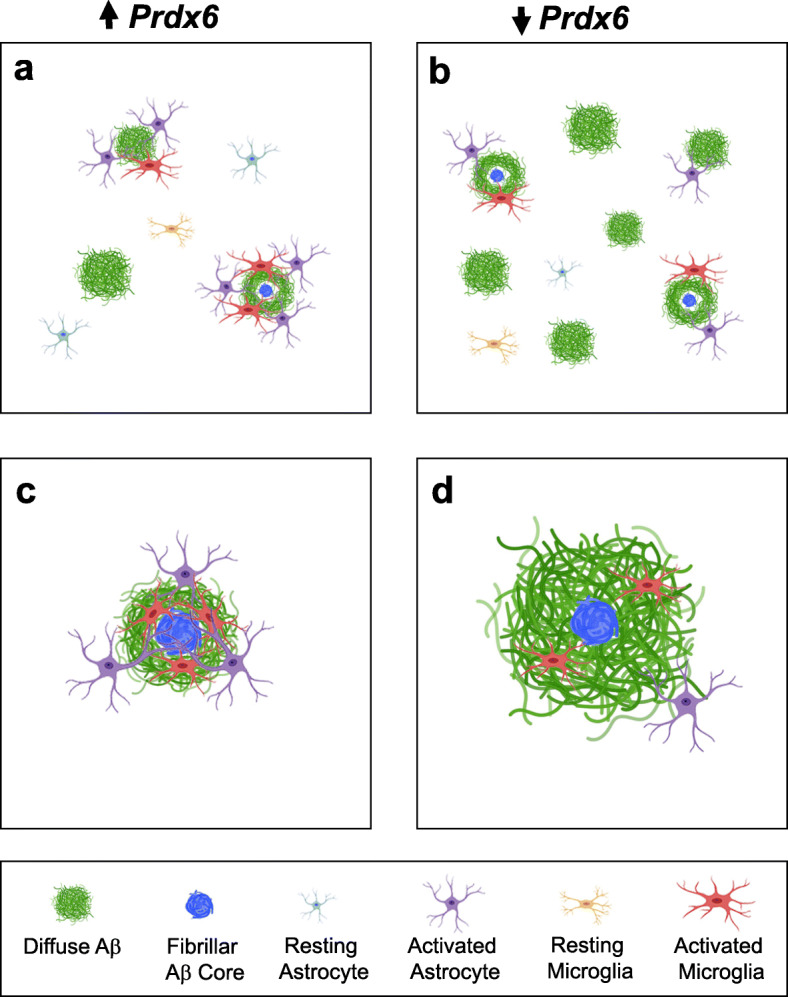
Schematic depiction of PRDX6 dependent mechanisms in Aβ homeostasis: **a** and **b** preventive effect on nascent plaque seeding and **c** and **d** remodeling of mature plaques. **a** Overexpression of *Prdx6* is associated with reduced number of nascent plaques and specifically those, which are devoid of activated astrocytes and/or microglia cells. **b** In contrast, *Prdx6* haplodeficiency increases number of nascent plaques including those, which are not associated with astro- and micro- glial cells. **c** Mature plaques in *Prdx6* overexpressing mice show increased plaque compactness and diminished deposition of diffuse Aβ peptide around the plaque fibrillar core. **d** Conversely, *Prdx6* haplodeficiency is associated with reduced plaque compactness and increased amount of diffuse Aβ in the plaque periphery. There is a direct association between the *Prdx6* gene dose and periplaque activation of astrocytes and microglia. Since *PRDX6* is an astrocytic protein these observations imply phagocytic function of microglia in Aβ proteostasis is regulated by astrocytes

Unlike microglia, function of astrocytes in Aβ proteostasis has received limited attention and remains of rudimentary understanding [38]. Peri-plaque astrocytic activation has been classically viewed as a passive response to neuritic degeneration through formation of a glial scar [39]. Here, we provide novel evidence that astrocytes play an active role in countering Aβ deposition, and identify PRDX6 protein as a key element of this process. PRDX6 is expressed by astrocytes but no other type of CNS glial cells [8, 9]. As a prerequisite for this study, we determined that two-fold change in *Prdx6* expression level does not affect astrocyte-resting state or alter soluble Aβ levels in AD Tg mice prior to appearance of Aβ plaques. Altered *Prdx6* expression also does not influence mounting of Aβ-associated astrogliosis, as the load of GFAP^+^ astrocytes remains proportional to that of fibrillar Aβ plaques. However, we find a striking, direct dependence between *Prdx6* gene dose and propensity of astrocytes to engage and penetrate Aβ plaques. We also show that *Prdx6* gene overexpression is associated with reduced Aβ plaque load through suppression of nascent plaque seeding and remodeling of mature plaque by phagocytically activated microglia, while *Prdx6* haplodeficiency conversely effects increased Aβ load in association with reduced periplaque microglia activation (Fig. 10 a-d). Demonstration that altering expression of an astrocytic inherent protein produces coextensive effect on periplaque microglia implies that astrocytes circuitously guide microglia dependent Aβ plaque processing.

PRDX6 is a cytoplasmic, enzymatic protein with independent Gpx and PLA_2_ activities [40]. Gpx activity is endowed by ^Cys^47, while the PLA_2_ activity by the triad of ^Ser^32, ^His^26, and ^Asp^140 brought into a single catalytic site by the tertiary PRDX6 folding [41]. PRDX6 is abundant in lung epithelium, endothelium, hepatocytes, and peripheral macrophages [5], where it is involved in antioxidant defense (with H_2_O_2_, short chain hydroperoxides, and phospholipid hydroperoxides as reduction substrates), phospholipid turnover, and cellular signaling [5]. In particular, PRDX6 is recognized for its ability to restitute peroxidized cell membrane lipids by their reduction through Gpx activity or excision through PLA_2_ action with subsequent replacement by PLA_2_-conjugated lysophosphatidylcholine acyl transferase activity. This particular functionality of PRDX6 is essential for the integrity of lung epithelium endowing resistance to constant oxidative damage [5, 6]. In AD, Aβ plaques constitute a source of locally confined yet enduring oxidative stress, which contributes importantly to disease pathogenesis by rendering protein misfolding, impairing ability of microglia to phagocytose Aβ while promoting their pro-inflammatory phenotype, and effecting neuritic degeneration [42]. Astrocytes, which penetrate Aβ plaques need to withstand consequences of chronic oxidative stress. In this study we showed that penetrance of Aβ plaques by astrocytes is associated with upregulation of PRDX6 protein expression along with redistribution of PRDX6 from the cell body to astrocytic processes. Similar reactive behavior of PRDX6 is known from extra-CNS cell lineages where oxidative stress upregulates PRDX6 level and promotes its translocation from the cytosol to the cell membrane allowing access to peroxidized lipids as the metabolic substrate [43]. In our previous work, we experimentally proved that PRDX6 renders astrocytes resistant to oxidative damage [8]. Astrocytes cultured from *Prdx6* overexpressing mice exhibit increased resistance to in vitro oxidative insult while astrocytes cultured from *Prdx6*^*−/−*^ mice, conversely show significantly reduced viability. Thus, greater penetrance of Aβ plaques by astrocytes in *APP/Prdx6*^*Tg*^ mice and reduced penetrance in *APP/Prdx6*^*+/−*^ mice reflect increased and decreased resistance of astrocytes to plaque-associated oxidative stress, respectively. Interestingly, we also observed that PRDX6 overexpressing astrocytes, which engage Aβ plaques have significantly reduced expression of C3, which is a marker of astrocytic neurotoxic phenotype, also known as A1 [33–35]. Conversely, in *Prdx6 haplodeficient* mice, which cannot mount proper PRDX6 expression, astrocytes not only poorly penetrate Aβ plaques but they also show upregulation of C3 expression. These results imply that chronic oxidative stress may contribute importantly to astrocytes assuming neurotoxic phenotypes, and that PRDX6 besides increasing endurance to oxidative insult helps in maintaining astrocytic homeostasis.

PRDX6 also is widely recognized for cellular signaling owing to its PLA_2_ activity, and this functionality is triggered by various cellular stressors requiring oxidative, inflammatory, and mechanical stress response [5, 7]. PRDX6/PLA_2_ activity can engage several signaling pathways including down-stream activation of NADPH oxidase 2 (NOX2), which is a member of the NOX/DuOX enzyme family generating O_2_^•-^ or H_2_O_2_ [44, 45]. Although rapid and uncontrollable release of O_2_^•-^ or H_2_O_2_ is a culprit in tissue injury during acute oxidative stress, low levels of either intracellular or extracellular O_2_^•-^ or H_2_O_2_ messengers are deemed essential for many homeostatic processes, involving intracellular signaling, microbial defense and guiding immune cell chemotaxis into sites of inflammation [46–50]. A well-established example of such, is NOX2-dependent activation of toll-like receptor (TLR) pathway promoting antibacterial autophagy [51]. Other examples of PLA_2_-dependent signaling include arachidonic acid pathway [52] and nuclear factor kappa B (NF-κB) pathway [53], which are up and downregulated by PRDX6 level, respectively. In summary, we hypothesize that the beneficence of PRDX6 overexpression against Aβ pathology is owed both to its anti-oxidative function, which increases oxidative stress endurance and allows for a more robust penetration of Aβ deposits by astrocytes and for the signaling function of PLA_2_ by which astrocytes circuitously can modulate the activity of plaque-associated microglia. It also is important to conceptualize, that with their somas located outside Aβ plaques and a substantial number of their processes spreading outward into the brain parenchyma, activated astrocytes are well-poised to extent signaling message far distant from the perimeter of Aβ deposits they engage. As the role of astrocytic PRDX6/PLA_2_ in cellular signaling remains unexplored, ours is the first observation to suggest it may be involved in astro/microglia crosstalk. Certainly, aforementioned PRDX6/PLA_2_-related signaling pathways like TLR and NF-κB are regarded important to microglia function [54–56]. Thus, in the future studies it will be imperative to precisely determine, which of these pathways underlie astrocyte/microglia cooperative effort to counter Aβ deposition.

Incipient stages of Aβ deposition remain underexplored. It is unclear how nascent Aβ plaques become recognized by glial cells and what sequence of events leads to periplaque glia activation. In the CNS, astrocytes numerically surpass microglia and form a dense surveying network readily responding to changes affecting milieu of the nervous tissue. Examining early stage of Aβ deposition in 6-month-old mice, we found that the numerical density of nascent plaques and the subset of these plaques devoid of astrocytes and microglia companionship vary inversely with the *Prdx6* gene dose. We also found that phagocytic activity of microglia associated with nascent plaques remains directly proportional to the *Prdx6* expression. These observations merit two conclusions. Firstly, that PRDX6 is involved in engagement of nascent plaques by co-operative action of astrocytes and microglia and secondly, that a portion of newly seeded plaques is cleared by activated microglia. Though the second notion may appear speculative, it is supported by the fact that the level of *APP* transcript and the level of soluble Aβ prior to the onset of Aβ deposition are similar across all three analyzed transgenic mouse lines rendering them similarly capable of plaque seeding. Our histopathological analysis also endorses recently published spatio-temporal transcriptomic studies in AD Tg mice, which indicate transcriptome alterations in astrocytes and microglia occur in parallel and coincide with emergence of early Aβ deposits [57, 58]. Thus, both studies emphasize an importance of astro/microglia cooperative effort in maintaining Aβ homeostasis.

Likewise, mature plaques we examined in mice aged 10 months reveal influence of *Prdx6* gene dosage on their morphology and degree of periplaque microglia activity. Aβ plaques in *Prdx6* overexpressing mice feature reduced amount of diffuse Aβ in the plaque brim, increased plaque compactness, and increased number and phagocytic activity of plaque-associated microglia. Such morphological changes reflect established function of microglia, known to phagocytose Aβ peptide in the plaque brim what limits plaque diffusion and facilitates compacting Aβ within the fibrillar core [36]. In *Prdx6* hemizygous mice Aβ plaques exhibit opposite features including increased amount of loose, non-fibrillar Aβ in the plaque brim and reduced plaque/core ratio indicative of reduced plaque compactness. Diminished number of periplaque microglial cells and microglia phagocytosis markers remain consistent with this more diffuse plaque morphology, resembling plaques in AD mice with *Trem2* hemizygocity or those harboring *Trem2* R47H mutant, both of which confer loss of TREM2 function and impair Aβ proteostatic function of microglia [29]. In fact, in our study peri-plaque microglia in *Prdx6* hemizygous mice feature reduced TREM2 expression, while those in *Prdx6* overexpressing mice show TREM2 upregulation. TREM2 and the TREM2/mTOR signaling pathway are recognized for their critical role in promoting phagocytic activity of microglia and other cells of myeloid linage [4]. However, how TREM2 expression is triggered during microglia activation and how it is sustained effecting microglia phagocytic phenotype remains unclear [59]. TREM2 activity is suppressed by activation of the NF-κB [59] pathway, which also is inversely regulated by PRDX6 expression level [53]. TREM2 also is stimulated by a set of specific ligands, including apoE [4, 59], which plaque content directly correlates with PRDX6 expression. Hence, through cellular signaling and increased apoE plaque content PRDX6 can exert two-prong effect on promoting TREM2 activity in periplaque microglia.

Axons passing through diffuse Aβ in the plaque periphery undergo degeneration forming spherical bodies [36, 37] and we found neuritic degeneration to be another essential feature directly associated with *Prdx6* gene dose. There is reduced numerical density of neuritic plaques in *Prdx6* overexpressing mice and conversely their density is increased in *Prdx6* hemizygous animals. While, these differences can be explained by variable effect of *Prdx6* expression on plaque seeding and resulting discrepancies in plaque density, we also found dissimilarities in the amount of degenerating axons within individual plaques. These differences likely reflect differential effect of *Prdx6* gene dosage on phagocytic activity of periplaque microglia and resulting degree of plaque diffusion [36]. It is also possible that anti-oxidant activity of PRDX6 itself contributes to attenuating neuritic degeneration as this process is driven by oxidative stress [42]. However, PRDX6 is compartmentalized to the cytoplasm and its anti-oxidant activity takes place primarily intracellularly rather than it is known to exert significant extracellular effect [5].

While this study utilizes models of chronic neurodegeneration and its conclusions imply beneficence of PRDX6 overexpression in Aβ pathology, opposing findings were described using acute/subacute models of neurodegeneration. Yun et al. showed that intraventricular infusion of oligomeric Aβ_42_ in mice transgenically overexpressing *Prdx6* paradoxically exacerbates oxidative stress and metrics of neurodegeneration compared to wild type mice [60]. Similarly, the same group showed that administration of MPTP to *Prdx6* Tg mice exacerbate loss of dopaminergic neurons in the substantia nigra compared to wild type animals [61]. Results of both studies suggest that overexpression of PRDX6 may potentiate outcome of an oxidative insult in the CNS. However, studies concerning PRDX6 function in the lungs may provide insight into understanding these seemingly opposing outcomes. There is extensive literature to indicate opposing behavior of pulmonary PRDX6 during chronic vs. fulminant oxidative insult (reviewed in [5]). While PRDX6 is recognized for its essential role in maintaining the integrity of  cellular membranes in the pulmonary epithelium, which is exposed to chronical low-grade oxidative stress, in acute lung injury, (e.g. in the course of sepsis), over activation of PLA_2_/NOX2 signaling pathway may lead to uncontrolled generation of O2^•-^ or H_2_O_2_ and paradoxical exacerbation of lung injury. Based on the original observations made by Yun and colleagues, brain PRDX6 also may be capable of paradoxical amplification of oxidative stress related injury. However, since Aβ deposition is an inherently protracted phenomenon, associated with chronic, low-level oxidative stress, we elected to test the role of PRDX6 in this process by developing and examining novel *APP/PS1* Tg mice varying in *Prdx6* gene expression. In contrast to the model utilizing intraventricular infusion of preformed Aβ_1–42_ oligomers, we demonstrated beneficence of PRDX6 overexpression in Aβ deposition and associated neurodegeneration, which we also confirmed by showing opposing outcomes when PRDX6 expression was reduced.

Unlike other cell types, astrocytes remain strikingly under-investigated in AD-related research [38]. There are few reports tying astrocyte dysfunction to propagation of Aβ pathology. Deletion of *Gfap* and *vimentin* genes encoding intermediate filaments of astrocytic cytoskeleton was shown to impair migration of astrocytes toward Aβ plaques, which resulted in accelerated plaque pathogenesis and enhanced neuritic degeneration in AD Tg mice [62]. Also, astrocytic rarefaction of Aβ plaques was identified as one of the neuropathological hallmarks in rapidly progressing AD, which is a rare but aggressive AD variant. Proteomic analysis in rapidly progressing AD identified PRDX6 as one of top proteins, whose peri-plaque expression is significantly reduced compared to sporadic disease [63]. In the backdrop of these observations, our study provides original insights into a role of astrocytes in Aβ proteostasis by proposing that astrocytes rather than being passive by-standers actively recognize Aβ deposits and circuitously modulate microglia phagocytic activity to counter Aβ accumulation. Plaques that escape initial seeding control and continue to grow remain penetrated by astrocytes, which resistance to oxidative  stress is mediated by PRDX6 antioxidant capacity. Astrocytes also propagate phagocytic activity of microglia, which limits plaque diffusion and curtails neuritic degeneration and this effect may be endowed by PLA_2_ signaling functionality of PRDX6. Thus, emerging evidence recognizes astrocytes as important players in Aβ proteostasis, and suggests that upregulation of PRDX6 expression can be of therapeutic benefit in AD.

## Conclusions

Our work identifies PRDX6 as an important factor selectively regulating periplaque activation of astrocytes and playing a critical role in astro/microglia cross talk in maintaining Aβ proteostasis.

## Supplementary information

**Additional file 1: Figure S1.** Comparative analysis of Aβ plaque load in female and male littermates shows significant opposing effects of *Prdx6* overexpression and haplodeficiency in both sexes. **Figure S2**. Segmentation analysis of LSCM images of Aβ plaques. **Figure S3** Overexpression of *Prdx6* is associated with increased accumulation of apoE within Aβplaques, while *Prdx6* haplodeficiency conversely reduces apoE plaque content. **Figure S4**. Expression of complement component 3 (C3) by plaque associated astrocytes varies inversely with *Prdx6* gene dose. **Figure S5.** Development of Aβ pathology is associated with upregulation of PRDX6 protein expression in astrocytes. **Figure S6.** Analysis of *Prdx6* RNA level in microglia cells. **Figure S7.** Phagocytic activity of microglia cells surrounding nascent plaques varies directly with *Prdx6* gene dose. Figure S8. *Prdx6* gene dose affects early stage of Aβ deposition. Supplementary methods. **Table S1.** Two-way ANOVA analysis of fibrillar and Aβ plaque load in the brain cortex and in the hippocampus in 10-month-old female and male littermates from *APP/Prdx6*^*Tg*^, *APP/Prdx6*^*+/+*^, and *APP/Prdx6*^*+/-*^ transgenic mouse lines.

## Data Availability

Raw images and datasets that support the findings of this study are available from the corresponding author upon reasonable request.

## Abbreviations

Aβ: β-amyloid
AD: Alzheimer’s disease
APP: Amyloid precursor protein
CD68: Cluster of differentiation 68
CNS: Central nervous system
GFAP: Glial fibrillary acidic protein
Gpx: Glutathione peroxidase
Iba1: Ionized calcium binding adaptor molecule
PLA_2_: Phospholipase A2
*Prdx6*: Peroxiredoxin 6 gene
PRDX6: Peroxiredoxin 6 protein
PS1: Presenilin 1
Th-S: Thioflavin-S
TREM2: Triggering Receptor Expressed in Myeloid cells 2
