# Supplementary material for: Peroxiredoxin 6 mediates protective function of astrocytes in Aβ proteostasis
Source: Mol Neurodegener. 2020 Sep 9;15:50. doi: 10.1186/s13024-020-00401-8 (PMC7487614; doi:10.1186/s13024-020-00401-8)
Supplement: Supplementary file 1 — Additional file 1: Figure S1. Comparative analysis of Aβ plaque load in female and male littermates shows significant opposing effects of Prdx6 overexpression and haplodeficiency in both sexes. Figure S2. Segmentation analysis of LSCM images of Aβ plaques. Figure S3 Overexpression of Prdx6 is associated with increased accumulation of apoE within Aβplaques, while Prdx6 haplodeficiency conversely reduces apoE plaque content. Figure S4. Expression of complement component 3 (C3) by plaque associated astrocytes varies inversely with Prdx6 gene dose. Figure S5. Development of Aβ pathology is associated with upregulation of PRDX6 protein expression in astrocytes. Figure S6. Analysis of Prdx6 RNA level in microglia cells. Figure S7. Phagocytic activity of microglia cells surrounding nascent plaques varies directly with Prdx6 gene dose. Figure S8. Prdx6 gene dose affects early stage of Aβ deposition. Supplementary methods. Table S1. Two-way ANOVA analysis of fibrillar and Aβ plaque load in the brain cortex and in the hippocampus in 10-month-old female and male littermates from APP/Prdx6Tg, APP/Prdx6+/+, and APP/Prdx6+/- transgenic mouse lines. [file 13024_2020_401_MOESM1_ESM.pdf]

Supplementary information

Supplementary figure legend and supplementary data

Fig. S1 Comparative analysis of Aβ plaque load in female and male littermates shows significant opposing effects of *Prdx6* overexpression and haplodeficiency in both sexes. Shown is quantification of fibrillar (Thioflavin-S$^+$) plaque load in the brain cortex **a** and in the hippocampus **b,** and the total Aβ plaque load (mAb HJ3.4$^+$) in the brain cortex **c** and in the hippocampus **d** in female and male mice of indicated genotypes: *APP/Prdx6*^{Tg}, *APP/Prdx6*$^{+/+}$, and *APP/Prdx6*$^{+/-}$. Values for individual animals and the mean values for animal groups ± SEM are presented. There are 9-12 female mice and 6-8 male mice per genotype, per group in **a** and **b**, and 9-11 female mice and 4-8 male mice per genotype, per group in **c** and **d.** Values for female mice are the same values presented as the bar graphs in Fig. 2b, d. *$p < 0.05$, **$p < 0.01$, ***$p < 0.001$, and ****$p < 0.0001$ for comparison across various *Prdx6* genotypes for mice of the same sex (Holm-Sidak's post-hoc test); and ♦♦$p < 0.01$, and ♦♦♦♦$p < 0.0001$ for comparison between female and male mice of matching *Prdx6* genotypes (Holm-Sidak's post-hoc test). Results of two-way ANOVA analysis preceding the Holm-Sidak's post-hoc test are included in Table S1.

There is a significant reduction in the fibrillar and the total Aβ plaque load in the brain cortex and in the hippocampus both in female and male *APP/Prdx6*^{Tg} mice when compared to sex-matched *APP/Prdx6*$^{+/+}$ controls. Conversely, *APP/Prdx6*$^{+/-}$ mice of both sexes show increase in the fibrillar and the total Aβ plaque load compared to sex-matched *APP/Prdx6*$^{+/+}$ controls. When matched for *Prdx6* genotype, female mice have significantly higher fibrillar and total Aβ plaque load in the brain cortex and in the hippocampus. Two-way ANOVA results summarized in Table S1, demonstrate significance of the main effect of sex and the main effect of *Prdx6* genotype for all measures.

Significant interaction between the main effects is noted for the fibrillar plaque load in the brain cortex and the total Aβ plaque load in the hippocampus. Overall, these findings demonstrate that *Prdx6* gene dose inversely modulates Aβ deposition both in female and male mice, but when matched for *Prdx6* genotype the load of Aβ plaque is invariably higher in females irrespective of plaque type and brain structure analyzed.

Fig. S2 Segmentation analysis of LSCM images of Aβ plaques. **a** A representative Aβ plaque from *APP/Prdx6*$^{+/+}$ mice (also used in Fig. 4) triple labeled with X-34 (fibrillar core), anti-Aβ (clone HJ3.4), and anti-GFAP antibodies. A collapsed stack of LSCM tomograms taken at the level of plaque core is presented. **b** Shown is separation of individual LSCM acquisition channels. Edges of Aβ$^+$ label are manually contoured to create the Aβ plaque mask. The mask is then transferred into the other channels to segment remaining components of the plaque. **c** Shown are black and white images of segmented plaque components as used for ensuing quantitative analysis. Aβ plaque cross-section area is defined as the area within the outline of Aβ plaque mask. “Plaque / core ratio” is calculated by dividing the Aβ plaque cross-section area by the area of X-34 label within the mask, while “GFAP$^+$ % plaque area” is the ratio of segmented GFAP$^+$ label to Aβ plaque cross-section area. Scale bar: 10 μm in **a**, **b**, and **c**

Fig. S3 Overexpression of *Prdx6* is associated with increased accumulation of apoE within Aβ plaques, while *Prdx6* haplodeficiency conversely reduces apoE plaque content. **a** Shown are epifluorescent microscopy images of representative Aβ plaques co-labeled with anti-Aβ and anti-apoE antibodies, and with X-34 (fibrillar core) from 10-month-old female mice of indicated genotypes. **b** Shown is quantitative analysis of the apoE plaque content. Ratio between the area of apoE$^+$ label to the area of Aβ$^+$ label (ApoE$^+$ % plaque area) was quantified in 30 plaques per genotype, and presented as the mean value + SEM. In *APP/Prdx6*^{Tg} mice the ApoE$^+$ plaque

coverage increases to 71.2% ($p < 0.0001$) from 50.6% in *APP/Prdx6*$^{+/+}$ controls, while in *APP/Prdx6*$^{+/-}$ mice it is reduced to 37.6% ($p < 0.0001$). **b** $p < 0.0001$ (ANOVA); ****$p < 0.0001$ (Holm-Sidak's post hoc test). Scale bar: 20 μm in **a**

Fig. S4 Expression of complement component 3 (C3) by plaque associated astrocytes varies inversely with *Prdx6* gene dose. **a** Shown are representative epifluorescent microscopy images of astrocytic clusters surrounding X-34^{+} amyloid core of Aβ plaques in the brain cortex from 10-month-old female mice of indicated genotypes. The sections are triple-labeled with X-34 and with anti-C3 and anti-GFAP antibodies. The microphotographs evidence reduced C3^{+} immunoreactivity in periplaque astrocytes in *APP/Prdx6*^{Tg} mice compared to *APP/Prdx6*$^{+/+}$ controls, while in *APP/Prdx6*$^{+/-}$ mice the C3^{+} immunoreactivity is increased. **b** Shown is quantitative analysis of the C3^{+} / GFAP^{+} Index, which was measured in female mice in 30 randomly selected plaques per genotype and presented as the mean + SEM. In *APP/Prdx6*^{Tg} mice the index is reduced by 26.7% compared to *APP/Prdx6*$^{+/+}$ controls ($p < 0.0001$), while in *APP/Prdx6*$^{+/-}$ mice it is increased by 31.8% ($p < 0.0001$). In direct comparison with the *APP/Prdx6*^{Tg} line, APP*/Prdx6*$^{+/-}$ mice show 1.8-fold higher value of the C3^{+} / GFAP^{+} Index ($p < 0.0001$). **b** $p < 0.0001$ (ANOVA); ****$p < 0.0001$ (Holm-Sidak's post hoc test). Scale bar: 20 μm in **a**

Fig. S5 Development of Aβ pathology is associated with upregulation of PRDX6 protein expression in astrocytes. Shown are representative LSCM images of hippocampal astrocytes from 3-month-old female mice **a**, and from 10-month-old female mice **b** of indicated genotypes. **c** Also shown are representative, low power, epifluorescent microscopy images of astrocytic clusters associated with Aβ plaques (arrows) and activated astrocytes associated with brain vessels (arrowheads) from the brain cortex of 10-month-old mice of indicated genotypes. All

sections are triple-labeled with X-34 (fibrillar Aβ), and with anti-PRDX6, and anti-GFAP antibodies. The photographs evidence the following gradient of PRDX6 expression across the genotypes: *APP/Prdx6*^{Tg} > *APP/Prdx6*$^{+/+}$ > *APP/Prdx6*$^{+/-}$. This gradient is apparent both in mice aged 3 months in **a** and those aged 10 months in **b** and **c**, which represent animals before the onset of Aβ deposition and those in which Aβ plaques are present, respectively. There is marked upregulation of PRDX6 expression in astrocytes associated with Aβ plaques in 10-month-old mice compared to 3-month-old animals for matching genotypes. LSCM images in **b** also reveal that upregulation of PRDX6 expression is associated with the spread of PRDX6 immunoreactivity from the soma to astrocytic processes. This effect is enhanced in astrocytes from *APP/Prdx6*^{Tg} mice, while in *APP/Prdx6*$^{+/-}$ mice it is nearly absent. The photographs in **c** also evidence expression of PRDX6 by activated, perivascular astrocytes. The expression level of PRDX6 protein by perivascular astrocytes is commensurate to the *Prdx6* gene dose across mouse lines. Perivascular astrocytic activation prior to the onset of Aβ deposition was not observed in any of the mouse lines (not shown).

Fig. S6 Analysis of *Prdx6* RNA level in microglia cells. Levels of *Prdx6* RNA were analyzed using real-time quantitative PCR and $2^{-\Delta\Delta Ct}$ method in primary cultures of wild type astrocytes (*Prdx6*$^{+/+}$), those of *Prdx6*$^{-/-}$ astrocytes, and of wild-type microglia (*Prdx6*$^{+/+}$), which were purified using anti-CD11b magnetic microbeads. Microglia cultures were additionally stimulated with lipopolysaccharides (LPSs) (1μg/mL) for 18 and 48 hr. Shown are mRNA levels expressed relative to those in wild-type (*Prdx6*$^{+/+}$) astrocytes and presented as mean values + SEM from 2-4 independent experiments, each using three technical replicates. $p < 0.0001$ (ANOVA); ****$p < 0.0001$ vs. *Prdx6*$^{+/+}$ astrocytes (Holm-Sidak's post hoc test). Relationships, which are not statistically significant, are not indicated on the graph.

Although *Prdx6* RNA can be detected in microglia cultured *in vitro*, its level represents merely 1% of that in astrocytes and does not increase with LPS stimulation. Results of this experiment remain consistent with negative anti-PRDX6 immunostaining of microglia in the brain parenchyma including microglia, which undergoes phagocytic activation around Aβ plaques.

Fig. S7 Phagocytic activity of microglia cells surrounding nascent plaques varies directly with *Prdx6* gene dose. **a** Shown are representative, epifluorescent microscopy images of nascent plaques co-labeled with X-34 and anti-CD68 antibody from female mice aged 6 months of indicated genotypes. The microphotographs evidence increase in $CD68^+$ immunostaining around the $X\text{-}34^+$ amyloid core in *APP/Prdx6*^{Tg} mice compared to *APP/Prdx6*$^{+/+}$ controls, while in *APP/Prdx6*$^{+/-}$ mice the $CD68^+$ immunoreactivity is reduced. **b** Shown is analysis of $CD68^+$ / $X\text{-}34^+$ Index for nascent plaques in the brain cortex of female mice. Values represent the mean value + SEM from 46-53 test areas per genotype. *APP/Prdx6*^{Tg} mice have 39.9% increase in the $CD68^+$ to $X\text{-}34^+$ ratio (Index) compared to *APP/Prdx6*$^{+/+}$ controls ($p < 0.0001$), while in *APP/Prdx6*$^{+/-}$ mice the Index is reduced by 20.1% ($p < 0.01$). In direct comparison with *APP/Prdx6*^{Tg} line, APP*/Prdx6*$^{+/-}$ mice have 1.8-fold lower value of the $CD68^+$ / $X\text{-}34^+$ Index ($p < 0.0001$). **b** $p < 0.0001$ (ANOVA); $^{**}p < 0.0001$, $^{****}p < 0.0001$ (Holm-Sidak's post hoc test). Scale bar: 10 μm in **a**

Fig. S8 *Prdx6* gene dose affects early stage of Aβ deposition. **a** Shown are representative microphotographs of coronal cross-sections through the somatosensory cortex and the dorsal hippocampus from female mice aged 6 months of indicated genotypes. Mice of this age represent an early stage of Aβ deposition, which in $APP_{SWE}/PS1_{dE9}$ model commences between the 5th and the 6th month of life. Sections are stained with Thioflavin-S (Th-S), which labels fibrillar Aβ within the plaques. Shown is quantification of Th-S^+ fibrillar plaque load **b** and fibrillar plaque numerical density **c** in the brain cortex and in the hippocampus in 6-month-old mice of indicated genotypes.

Values represent mean + SEM from 6 female mice per genotype. **b** and **c** $p < 0.0001$ in (ANOVA); *$p < 0.05$, **$p < 0.01$, and ****$p < 0.0001$ (Holm-Sidak's post hoc test). Abbreviations: Crtx – cortex, Hip – hippocampus. Scale bar: 500 μm in **a**

Fibrillar plaque load in *APP/Prdx6*^{Tg} mice in the brain cortex is reduced by 17.3% ($p < 0.01$) and by 20.3% in the hippocampus ($p < 0.05$) compared to *APP/Prdx6*$^{+/+}$ controls, while in *APP/Prdx6*$^{+/-}$ mice it is increased in respective structures by 22.7% ($p < 0.0001$) and 40.4% ($p < 0.0001$). *APP/Prdx6*^{Tg} mice also show significant reduction in the numerical density of fibrillar plaques by 12.2% in the brain cortex ($p < 0.05$) and by 22.0% in the hippocampus ($p < 0.05$) compared to *APP/Prdx6*$^{+/+}$ controls, while in *APP/Prdx6*$^{+/-}$ mice plaque density is increased by 30.0% ($p < 0.0001$) and by 25.5% ($p < 0.01$) in the brain cortex and in the hippocampus, respectively. In direct comparison with *APP/Prdx6*^{Tg} line, the fibrillar plaque load in 6-month-old female APP*/Prdx6*$^{+/-}$ mice is 1.5-fold higher in the brain cortex and 1.8-fold higher in the hippocampus ($p < 0.0001$), while the numerical density of fibrillar plaques is 1.5-fold higher ($p < 0.0001$) and 1.6-fold higher ($p < 0.0001$) in respective structures.

Supplementary methods

Quantitative analysis of the apoE plaque content

Free -floating coronal brain sections were pretreated with 10 mM sodium citrate and 0.05% Tween 20 (pH 6.0) for 20 min at 85°C [1], and then triple labeled with X-34, anti-A$\beta_{1\text{-}16}$ (rabbit polyclonal 1:500; BioLegend), and anti-apoE (mouse monoclonal, [clone HJ6.3] [2] 1:100; gift of DM Holtzman) primary antibodies followed by secondary antibodies: Alexa Fluor 488 conjugated donkey anti-rabbit (1:500; Jackson ImmunoResearch) and Alexa Fluor 594 conjugated donkey

anti-mouse (1:500; Jackson ImmunoResearch), respectively. Microphotographs of X-34/Aβ/apoE triple-labeled plaques were captured in the brain cortex under x40 objective using 80i Nikon fluorescent microscope equipped in monochrome DSQi1Mc camera and NIS Elements Imaging Software v. 4.13 (Nikon Corp., Tokyo, Japan). Areas of apoE and Aβ labels for each plaque were determined using NIH ImageJ v1.52 and divided by one another to obtain ApoE^{+} % plaque area. Ten plaques in the brain cortex per animal, from three females per genotype were randomly selected for analysis.

Analysis of C3 expression in plaque-associated astrocytes

Sodium citrate pretreated, free-floating brain sections were triple labeled with X-34, and anti-C3 (rat monoclonal 1:100; Hycult Biotech Inc., Germantown, PA) and anti-GFAP (rabbit polyclonal 1:2000; DAKO) primary antibodies followed by secondary antibodies: Alexa Fluor 594 conjugated goat anti-rat (1:500; Jackson ImmunoResearch) and Alexa Fluor 488 conjugated goat anti-rabbit (1:500; Jackson ImmunoResearch). C3 / GFAP co-labeled astrocytes surrounding the X-34^{+} core of Aβ plaques were photographed under x40 objective using 80i Nikon fluorescent microscope as described above. Captured images were analyzed using NIH ImageJ v1.52. Individual clusters of plaque-associated astrocytes were manually outlined on digitized images and the integrated densities for C3 and GFAP immunofluorescence were measured within the outlined area of interest as previously described [3]. C3^{+} / GFAP^{+} Index was calculated by dividing C3 integrated density by that of GFAP for the same plaque. Ten plaques in the brain cortex per animal, from three females per genotype were randomly selected for analysis.

Analysis of *Prdx6* RNA level in astrocytes and microglial cells *in vitro*

Mixed glia cultures were established from the brain cortex of P0-P3 pups as previously described [4] and maintained in Dulbecco's Modified Eagle Medium with F12 Nutrient Mixture (DMEM/F12)

(Thermo Fisher Scientific) supplemented with 10% fetal bovine serum (FBS), 5% horse serum, 1 mM sodium pyruvate, 100μg/mL streptomycin, and 100U/mL penicillin. At 18 day *in vitro* (DIV) the culture was treated with 60 mM Lidocaine in Ca^{2+}/Mg^{2+}-free Hanks' Balanced Salt Solution (Thermo Fisher Scientific) for 15 min to detach microglia cells [5]. The suspension of free-floating cells was collected and immediately centrifuged at 1,000 x g for 5 minutes in the presence of 50 μM EDTA. Resulting pellet was resuspended in a buffer containing 2.6 mM KCl, 137.9 mM NaCl, 1.4 mM KH_2PO_4, and 8.05 mM Na_2HPO_4 (pH 7.4) supplemented with 0.5% FBS. The cells were counted and the volume was adjusted to achieve cellular density of 1.1 x 10^8/mL. Aliquots of the cell suspension were incubated with magnetic micro beads coated with anti-CD11b antibody (9:1 vol/vol) (Miltenyi Biotec; Bergisch Gladbach, Germany) at room temperature for 15 minutes. $CD11b^+$ microglial cells were positively selected on the LS Column (Miltenyi Biotec) according to manufacturer provided manual, counted, and plated on poly-D-lysine coated 12-well plates in the amount of 4 x 10^5 cells per well. In the LPSs challenge experiment, micro-bead isolated microglial cells were cultured in DMEM/F12 with aforedescribed supplements for two days and then treated with 1μg/mL of LPSs for 18 or 48 hours. At the conclusion of each experiment, microglia cells were lysed and their RNA was extracted using RNeasy Mini Kit (Qiagen Sciences Inc.) following manufacturer provided manual.

Astrocytes, which remained in the original culture following lidocaine treatment were grown in DMEM/F12 supplemented with 10% FBS, 1 mM sodium pyruvate, 100 μg/mL streptomycin, 100 U/mL penicillin and 8 mM cytosine β-D-arabinoside to eliminate growth of non-astrocytic cells until 25 DIV. Then, they were lysed and their RNA was extracted using RNeasy Mini Kit.

Total RNA extracted from harvested cells was reverse transcribed into cDNA, and the level of *Prdx6* transcript was determined using qRT-PCR $2^{-\Delta\Delta Ct}$ method as described in the Material and Methods section. *Gapdh* gene was used as the calibrator for the $2^{-\Delta\Delta Ct}$ method.

Quantitative analysis of fibrillar plaque load and phagocytic activity of microglia in 6-month-old animals

The load of fibrillar Aβ plaques was analyzed in female mice aged 6 months on sections stained with Th-S for consistency with the results obtained in 10-month-old animals. The same whole-section approach to plaque load analysis was used as described in the Material and Methods section of the main manuscript. In addition, numerical density of fibrillar plaques on cross-sectional profiles of the brain cortex and the hippocampus was determined using NIH ImageJ v1.52.

Phagocytic activity of microglia was assessed by determining the $CD68^+$ / $X\text{-}34^+$ Index of nascent plaques. Free-floating brain sections were first pretreated with sodium citrate, as described above, and then stained with X-34 (10 mM in 40% ethanol [pH 10]), and subsequently immunostained with anti-CD68 rat monoclonal antibody(1:200; Abcam Inc.), which was followed by Alexa Fluor 594 conjugated goat anti-rat secondary antibody (1:500; Jackson ImmunoResearch). Three coronal sections from three brains randomly selected per genotype were used for analysis. The sections were taken at the approximated levels of the anterior commissure, the rostral portion of the hippocampus, and the mammillary bodies. The entire brain cortex on each section was photographed under x20 objective using 80i Nikon fluorescent microscope as described above which produced 5-6 non-overlapping, 0.44 mm^2 test areas per cortical profile. $X\text{-}34^+$ and $CD68^+$ loads were thresholded and quantified on each test area using NIH ImageJ v1.52. Since the analysis focused on the nascent plaques, infrequent mature plaques, arbitrary defined as having $X\text{-}34^+$ core > 40um^2 were edited out from digitized images. $CD68^+$ / $X\text{-}34^+$ Index was calculated by dividing the $CD68^+$ load by the $X\text{-}34^+$ load for each test area.

Supplementary references

1. Cheng-Hathaway PJ, Reed-Geaghan EG, Jay TR, Casali BT, Bemiller SM, Puntambekar SS, von Saucken VE, Williams RY, Karlo JC, Moutinho M, et al. The Trem2 R47H variant confers loss-of-function-like phenotypes in Alzheimer's disease. Mol Neurodegener. 2018;13:29.
2. Liao F, Zhang TJ, Jiang H, Lefton KB, Robinson GO, Vassar R, Sullivan PM, Holtzman DM. Murine versus human apolipoprotein E4: differential facilitation of and co-localization in cerebral amyloid angiopathy and amyloid plaques in APP transgenic mouse models. Acta Neuropathol Commun. 2015;3:70.
3. Pankiewicz JE, Sanchez S, Kirshenbaum K, Kascsak RB, Kascsak RJ, Sadowski MJ. Anti-prion Protein Antibody 6D11 Restores Cellular Proteostasis of Prion Protein Through Disrupting Recycling Propagation of PrP(Sc) and Targeting PrP(Sc) for Lysosomal Degradation. Mol Neurobiol. 2019;56:2073-2091.
4. Kuszczyk MA, Sanchez S, Pankiewicz J, Kim J, Duszczyk M, Guridi M, Asuni AA, Sullivan PM, Holtzman DM, Sadowski MJ. Blocking the Interaction between Apolipoprotein E and Abeta Reduces Intraneuronal Accumulation of Abeta and Inhibits Synaptic Degeneration. AmJ Pathol. 2013;182:1750-1768.
5. Bronstein R, Torres L, Nissen JC, Tsirka SE. Culturing microglia from the neonatal and adult central nervous system. J Vis Exp. 2013:50647.

Table S1. Two-way ANOVA analysis of fibrillar and Aβ plaque load in the brain cortex and in the hippocampus in 10-month-old female and male littermates from *APP/Prdx6*^{Tg}, *APP/Prdx6*$^{+/+}$, and *APP/Prdx6*$^{+/-}$ transgenic mouse lines.

Parameter	Main Effect of *Prdx6* Genotype	Main Effect of Sex	Main Effect Interaction
Fibrillar plaque load, Brain cortex	$F_{(2, 51)} = 119.7$; $p < 0.0001$	$F_{(1, 51)} = 256.7$; $p < 0.0001$	$F_{(2, 51)} = 8.831$; $p < 0.0005$
Fibrillar plaque load, Hippocampus	$F_{(2, 52)} = 64.99$; $p < 0.0001$	$F_{(1, 52)} = 104.7$; $p < 0.0001$	$F_{(2, 52)} = 3.095$; $p < 0.0537$
Aβ plaque load, Brain cortex	$F_{(2, 44)} = 161.4$; $p < 0.0001$	$F_{(1, 44)} = 151.4$; $p < 0.0001$	$F_{(2, 44)} = 1.872$; $p < 0.1659$
Aβ plaque load, Hippocampus	$F_{(2, 44)} = 169.3$; $p < 0.0001$	$F_{(1, 44)} = 206.5$; $p < 0.0001$	$F_{(2, 44)} = 33.11$; $p < 0.0001$

Figure S1

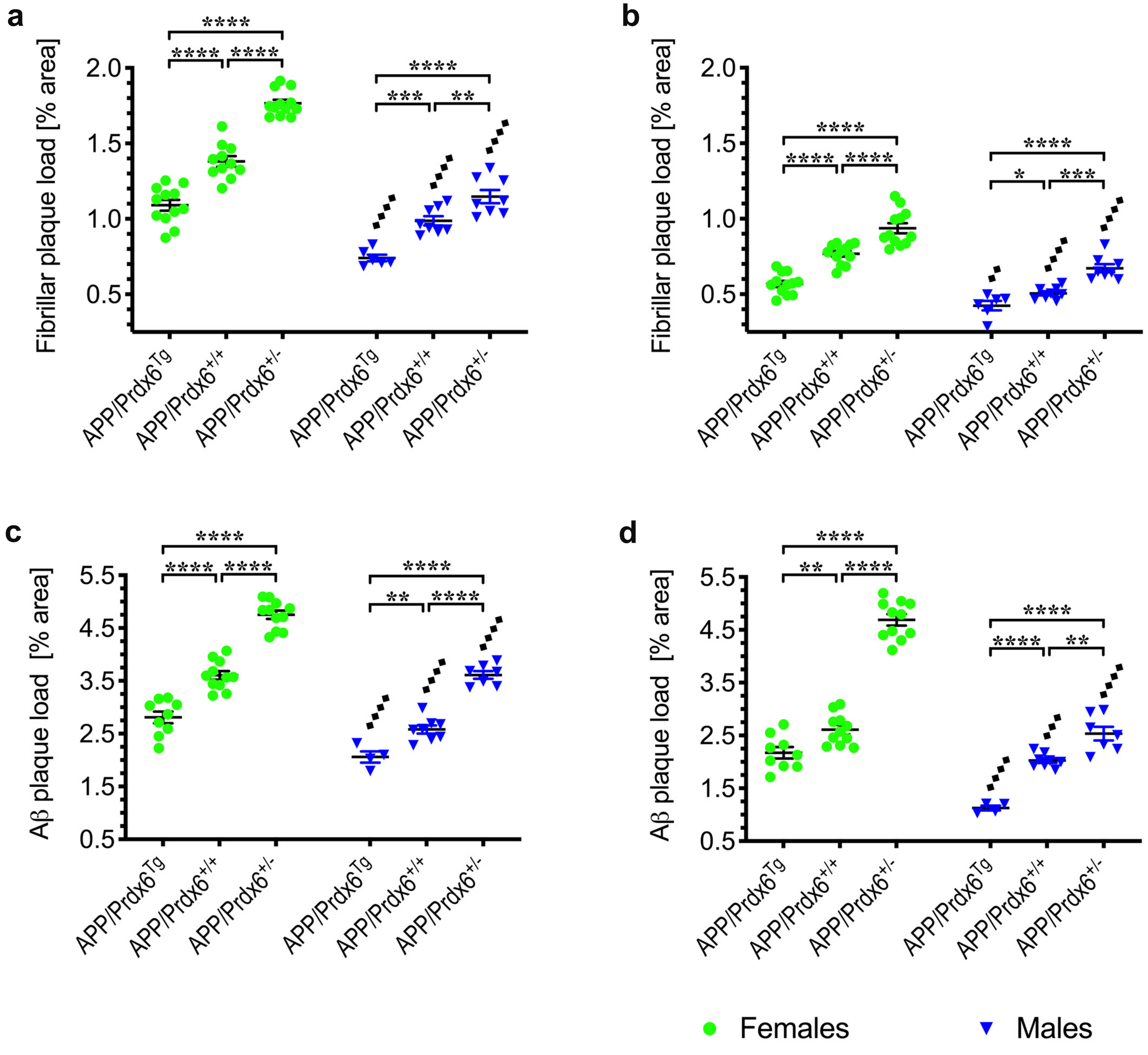
a
b
c
d
Fibrillar plaque load [% area]
Aβ plaque load [% area]
APP/Prdx6Tg
APP/Prdx6+/+
APP/Prdx6+/-
Females
Males

Figure S2

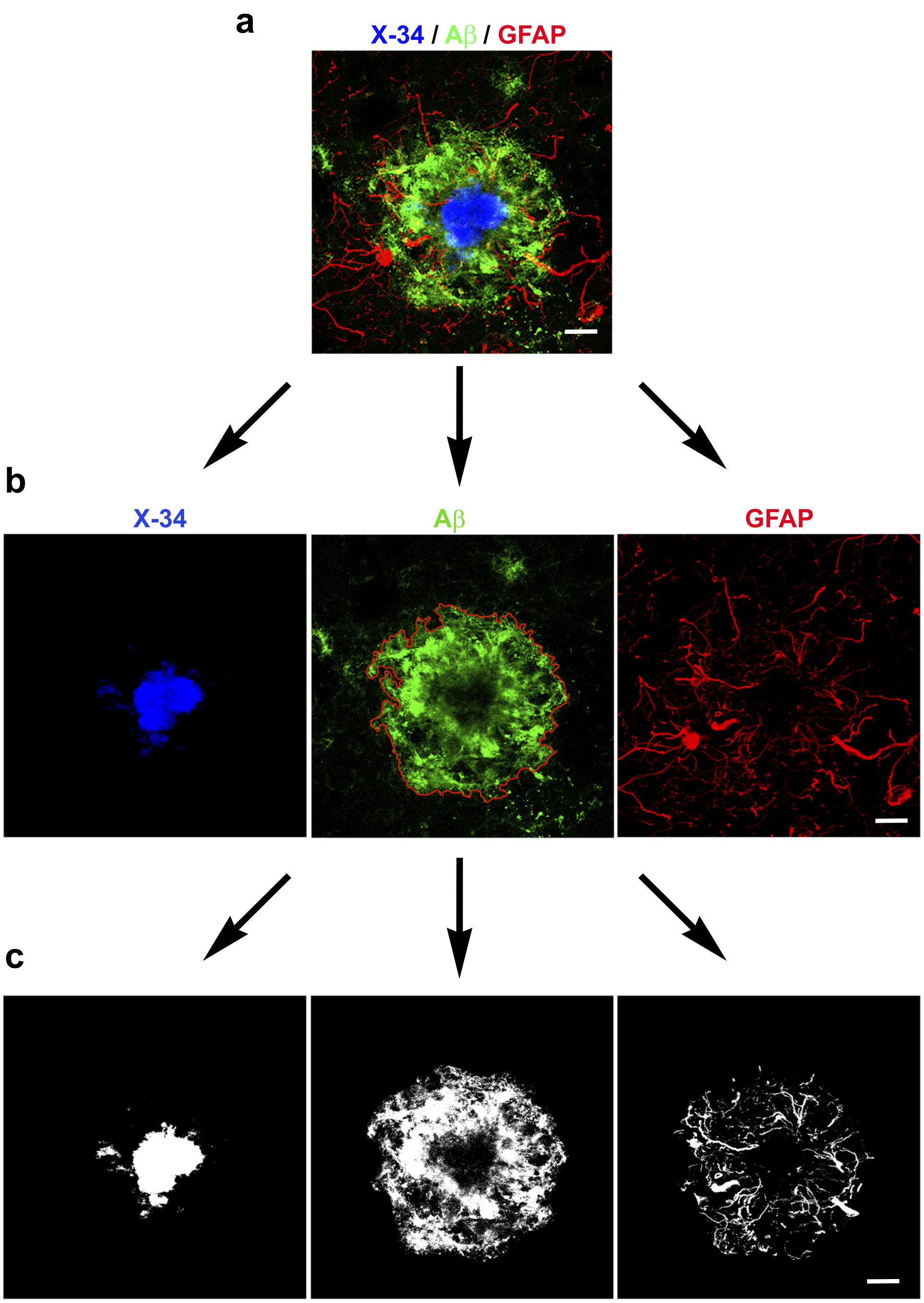

Figure S3

a

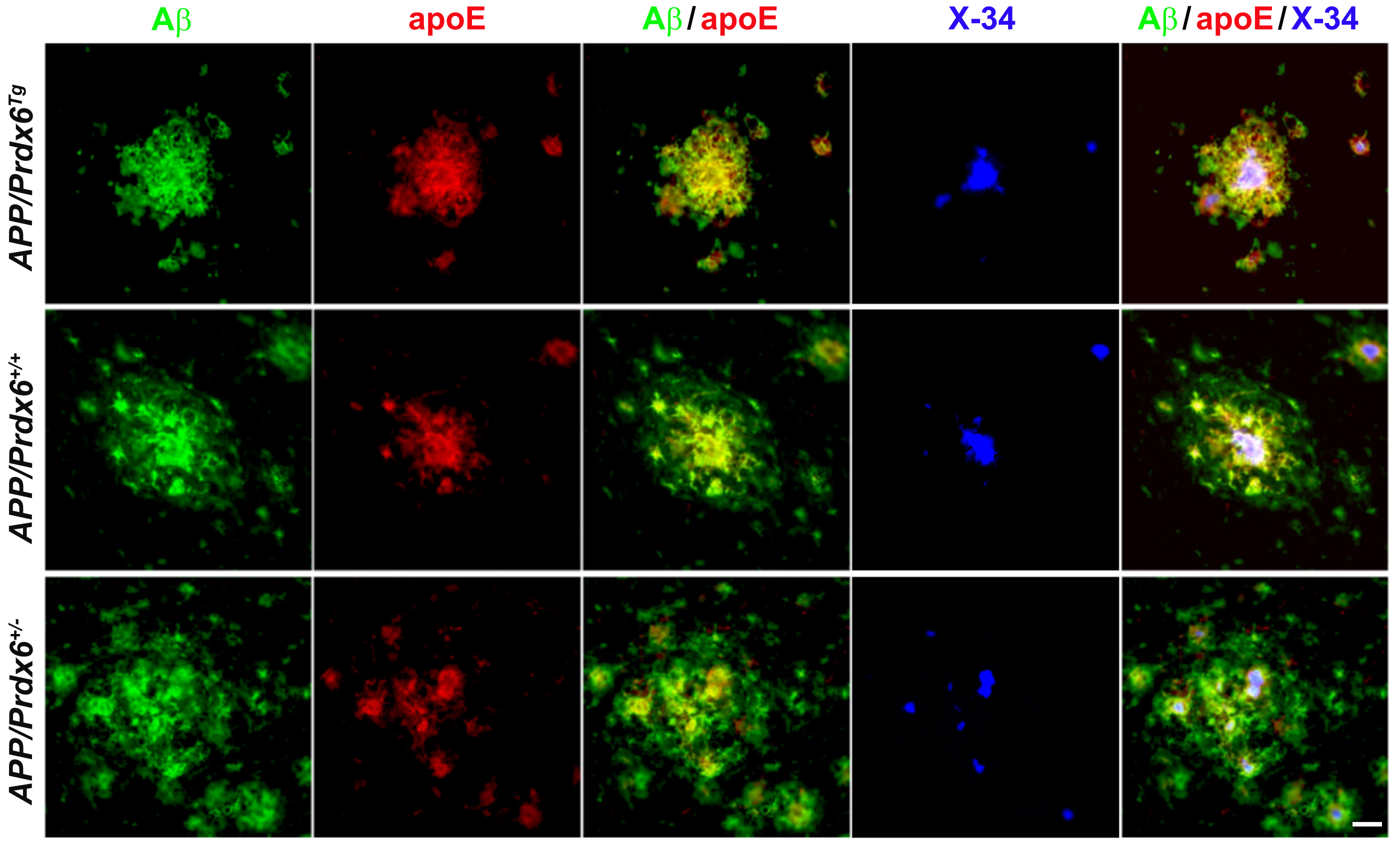

Aβ
apoE
Aβ / apoE
X-34
Aβ / apoE / X-34
APP/Prdx6Tg
APP/Prdx6+/+
APP/Prdx6+/-

b

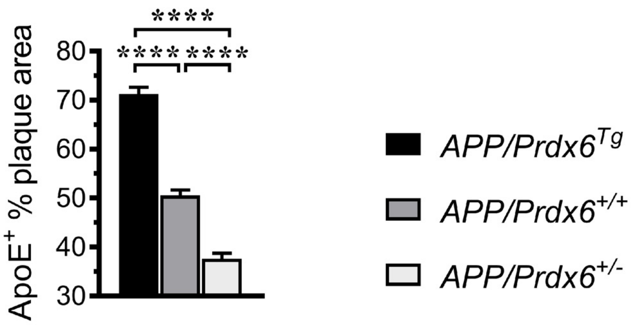

ApoE+ % plaque area
80
70
60
50
40
30
****
****
APP/Prdx6Tg
APP/Prdx6+/+
APP/Prdx6+/-

Figure S4

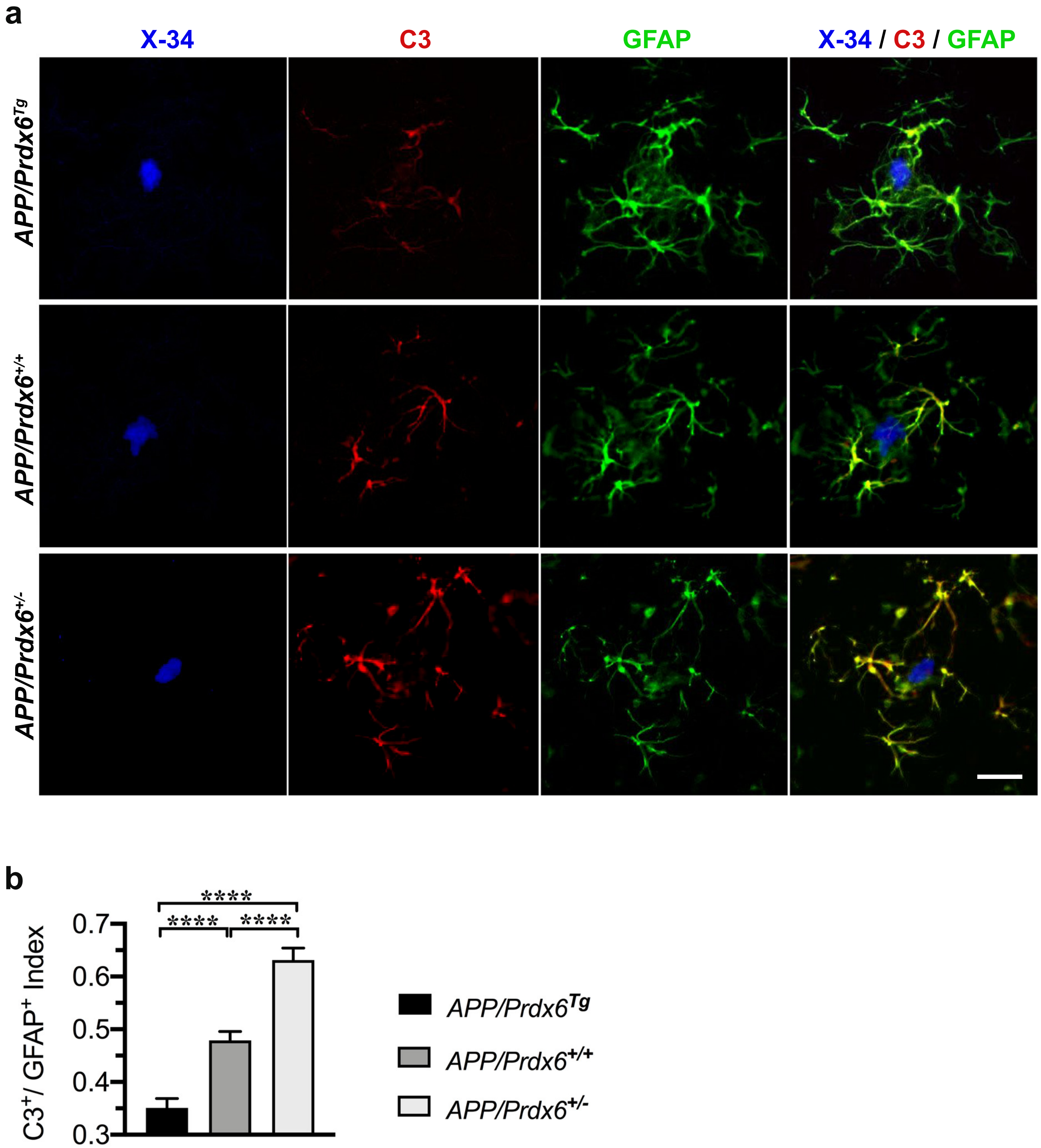

a
X-34
C3
GFAP
X-34 / C3 / GFAP
APP/Prdx6Tg
APP/Prdx6+/+
APP/Prdx6+/-
b
****
****
****
0.7
0.6
0.5
0.4
0.3
C3+/ GFAP+ Index
APP/Prdx6Tg
APP/Prdx6+/+
APP/Prdx6+/-

Figure S5

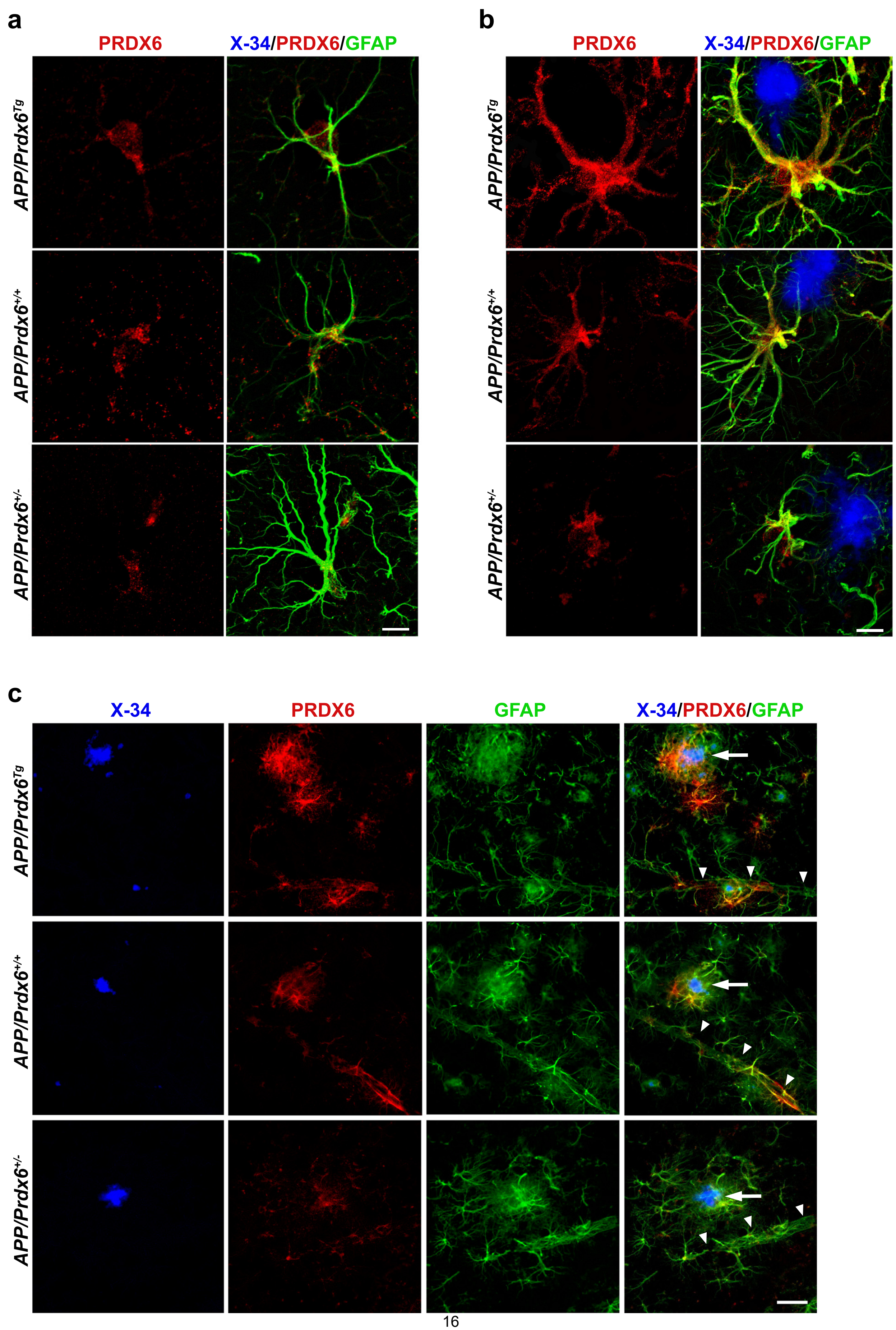
a
b
c
PRDX6
X-34/PRDX6/GFAP
X-34
GFAP
APP/Prdx6Tg
APP/Prdx6+/+
APP/Prdx6+/-

Figure S6

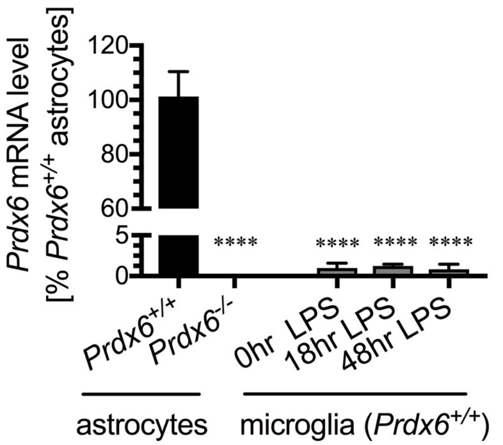

Prdx6 mRNA level
[% *Prdx6*+/+ astrocytes]
120
100
80
60
5
0
****
****
****
****
Prdx6+/+
Prdx6-/-
0hr LPS
18hr LPS
48hr LPS
astrocytes
microglia (*Prdx6*+/+)

Figure S7

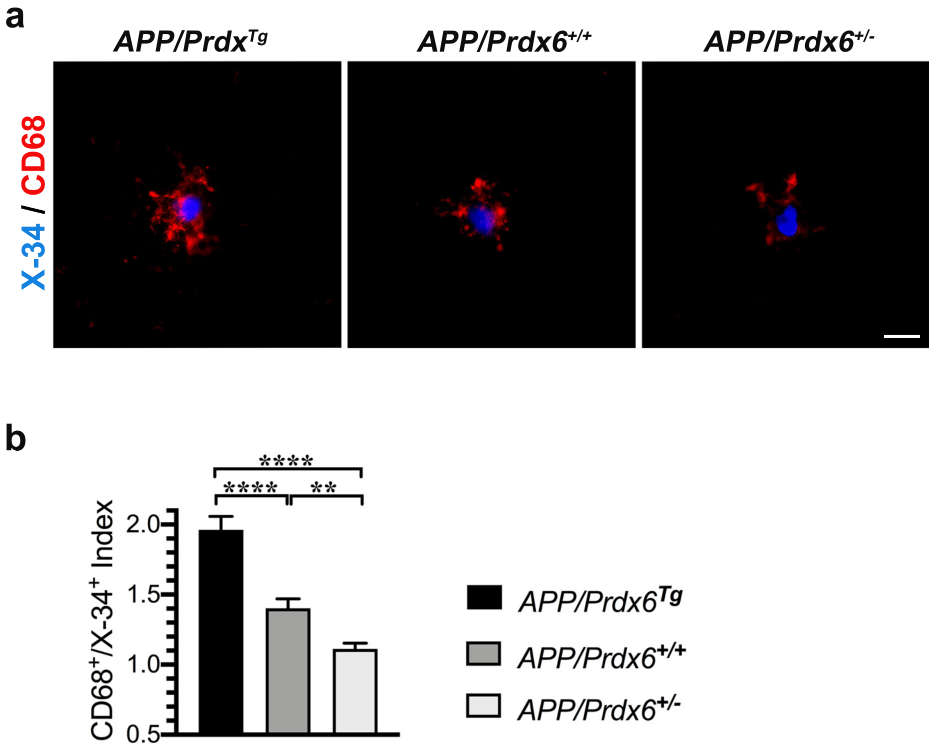
a
APP/PrdxTg
APP/Prdx6+/+
APP/Prdx6+/-
X-34 / CD68
b
****
****
**
CD68+/X-34+ Index
2.0
1.5
1.0
0.5
APP/Prdx6Tg
APP/Prdx6+/+
APP/Prdx6+/-

Figure S8

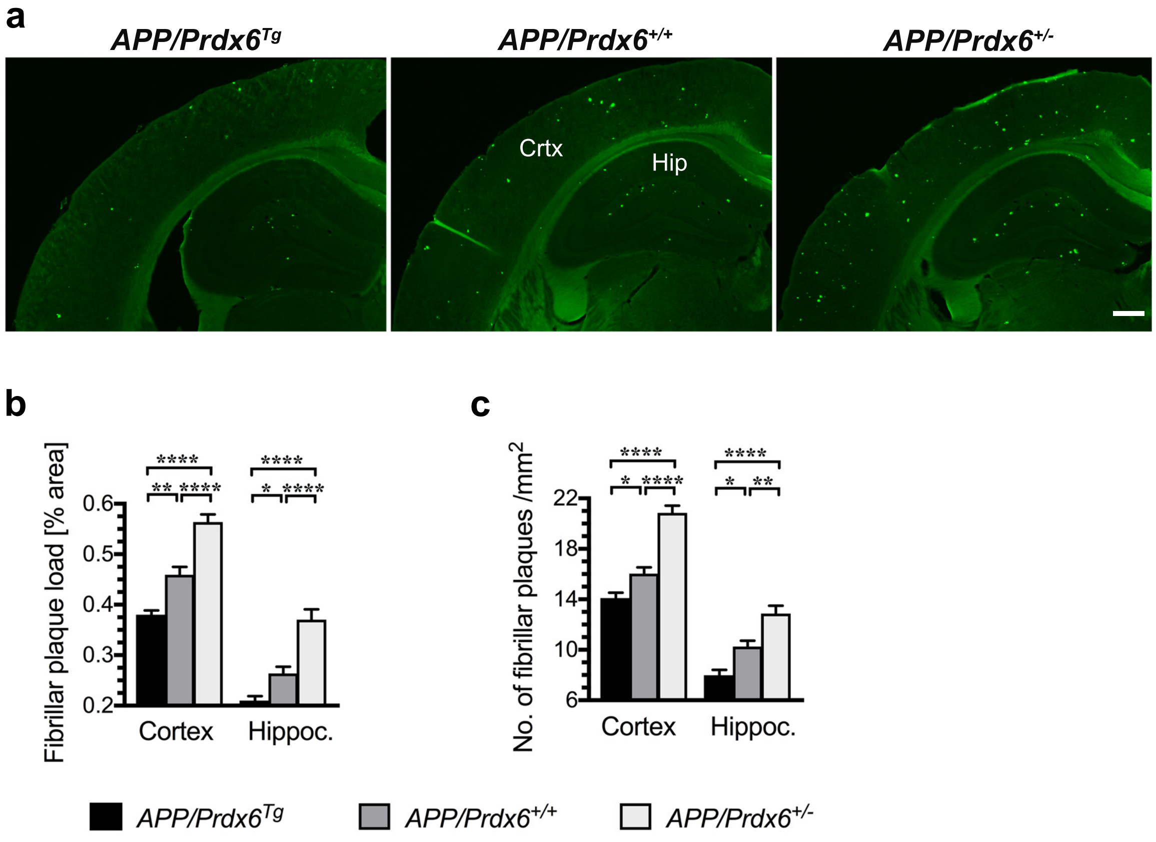

a
APP/Prdx6^Tg
APP/Prdx6^+/+
APP/Prdx6^+/-
Crtx
Hip
b
Fibrillar plaque load [% area]
0.6
0.5
0.4
0.3
0.2
Cortex
Hippoc.
c
No. of fibrillar plaques /mm²
22
18
14
10
6
Cortex
Hippoc.
APP/Prdx6^Tg
APP/Prdx6^+/+
APP/Prdx6^+/-
